# Reporting of post-operative rehabilitation interventions for Total knee arthroplasty: a scoping review

**DOI:** 10.1186/s12891-021-04460-w

**Published:** 2021-06-30

**Authors:** Nora Bakaa, Lu Hsi Chen, Lisa Carlesso, Julie Richardson, Luciana Macedo

**Affiliations:** School of Rehabilitation Sciences, Faculty of Health Sciences, McMaster University, Institute of Applied Health Sciences, Room 403, 1400 Main St. W., Hamilton, ON L8S 1C7 Canada

**Keywords:** TKA, Total knee arthroplasty, Exercise rehabilitation, CERT, Adherence, Scoping review

## Abstract

**Objective:**

The aim of this study was to evaluate the completeness of reporting of exercise adherence and exercise interventions delivered as part of clinical trials of post-operative total knee replacement (TKA) rehabilitation.

***Design: Scoping review***

**Literature search:**

A literature search was conducted in PubMed, EMBASE, AMED, CINAHL, SPORTDiscus and Cochrane Library.

**Study selection criteria:**

All randomized controlled trials (RCT) that examined post-operative exercise-based interventions for total knee arthroplasty were eligible for inclusion. Studies that were multifactorial or contained exercise interventions for both hip and knee arthroplasty were also included.

**Data synthesis:**

The definition, type of measurement used and outcome for exercise adherence were collected and analyzed descreptively. Quality of reporting of exercise interventions were assessed using the Consensus for Exercise Reporting Tool (CERT) and the Cochrane Risk of Bias Tool.

**Results:**

There were a total of 112 RCTs included in this review. The majority of RCTs (63%, *n* = 71) did not report exercise adherence. Only 23% (*n* = 15) of studies provided a definition of adherence. RCTs were of poor quality, with 85% (*n* = 95) of studies having high or unclear risk of bias. Reporting of exercise interventions was poor, with only 4 items (of 19) (21%) of the CERT adequately reported (88–99%), with other items not fulfilled on at least 60% of the RCTs. There were no RCTs that had fulfilled all the criteria for the CERT.

**Conclusion:**

The RCTs included in this study poorly reported exercise adherence, as well as description of the post-operative TKA rehabilitation intervention. Future RCTs should use valid and reliable measures of adherence and a proper tool for reporting of exercise interventions (e.g., CERT, TiDER).

**Pre-registration:**

***OSF:*** ***https://osf.io/9ku8a/***

**Supplementary Information:**

The online version contains supplementary material available at 10.1186/s12891-021-04460-w.

## Key points

1. Authors should use valid and reliable outcome measures of exercise adherence across all study treatment arms.
2. Authors should clearly define exercise adherence and use a continuous measure (rather than a cut-off) to avoid reporting bias.
3. In addition to using an RCT reporting guideline, authors should include an exercise reporting tool (e.g., TiDER or CERT).
4. Authors should adequately report exercise interventions even if it is an adjunct to the main intervention (e.g., electrotherapy plus exercise, control group, etc.).

## Background

Rehabilitation, specifically therapeutic exercise, is one of the most recommended interventions for improving health outcomes after total knee arthroplasty (TKA) [1]. A recent meta-analysis of 27 studies provided low-to-moderate quality evidence that exercise interventions (e.g. aerobic exercises, strength training) improved patient outcomes after TKA [2]. There was low quality evidence for pain reduction (SMD − 0.65, 95% CI [− 1.22, − 0.08]), and moderate quality evidence for improved physical function (SMD − 0.40, 95% CI [− 0.74, − 0.07) at 8-weeks follow-up [2]. While the benefits of exercise have been highlighted within the literature, studies that have assessed rehabilitation after TKA often lack detailed descriptions of the intervention (e.g. dosage, frequency, intensity, duration, etc.), thereby limiting translation to clinical practice [3–5].

Clinical guidelines for post-operative rehabilitation for TKA often do not provide detailed information about the recommended interventions, [6, 7] leading to variation in care and treatment approaches that are refined by the treating practitioners. A systematic review assessing rehabilitation guidelines for post-operative TKA concluded that proper instructions and adherence to rehabilitation can improve post-operative recovery and improve physical activity levels post-operatively [8]. Further, there is evidence to suggest that lack of exercise after TKA may lead to poor post-operative outcomes, including increased pain and functional disability [1]. However, there is very little evidence surrounding patient adherence to exercise recommendations after TKA, which may also impact the implementation of these interventions [8].

Exercise adherence can be defined as: “the extent to which individuals undertake prescribed behaviour accurately and at the agreed frequency, intensity and duration.” [9] Despite the importance of adherence to exercise interventions, there are no studies that focus on adherence to therapeutic exercise after TKA. In a recent systematic review, some studies that assessed effectiveness of exercise after TKA have reported adherence to exercise interventions, however, it is unclear how adherence is defined (e.g. compliance, concordance, etc.) or measured within a randomized controlled trial [5].

Therefore, the aim of this study was to evaluate completeness of reporting of exercise adherence and exercise interventions delivered as part of clinical trials of post-operative TKA rehabilitation. While systematic reviews evaluate a specific range of studies to answer focused questions of effectiveness, scoping reviews often have a broader mandate to examine the range and extent of research activity in a field [10]. In this study, a scoping review was used to answer the following questions:

1. How is exercise adherence measured and reported in post-operative TKA rehabilitation programs?
2. How is exercise adherence defined in post-operative TKA research?
3. How complete is the reporting of exercise interventions (e.g., frequency, intensity, etc.) in post-operative TKA rehabilitation?

## Methods

A scoping review was chosen for this study to examine the characteristics of exercise adherence and assess the quality of reporting of exercise intervention in post-operative TKA.

This scoping review followed the Preferred Reporting Items for Systematic Reviews and Meta Analyses (PRISMA) guidelines for scoping reviews [11]. We also used Arksey & O’Malley’s [12] methodological framework, modified by Levac et al., [10] to guide this scoping review. This framework included the following stages: 1) Identifying the research question; 2) Identifying relevant studies; 3) Selecting studies; 4) Charting the data, collating, and summarizing; and 5) Reporting the results. This protocol was pre-registered with OSF: https://osf.io/9ku8a/.

### Inclusion criteria for this review

#### Type of participants

Studies with adults (> 18 years old), that underwent bilateral or unilateral TKA, were included in this review.

#### Type of studies

All randomized controlled trials (RCT) that examined post-operative exercise-based interventions (home-based, inpatient, or outpatient) were eligible for inclusion. If the control group was not an exercise intervention, it was still included in this review. An exercise-based intervention was defined as: “an intervention that involved participants completing more than one session of physical exercises such as strengthening, flexibility, and/or aerobic activities.” [13] If a study was multifactorial (e.g., exercise and education), then the study was included if the primary intervention was exercise. Exercise was considered a primary intervention, if exercise was a major component through the description of the intervention as well as if the effectiveness of the interventions were evaluated using common exercise outcome measures (e.g., pain, function, patient satisfaction with exercise, etc.). If a study contained an exercise intervention for both hip and knee arthroplasty, it was included. No language restrictions were applied. Gray literature studies (e.g., abstracts, conferences, commentaries, editorials), systematic reviews, case studies, psychometric studies, studies where patients with total knee replacement were identified retrospectively were excluded. Studies that included passive forms of exercise (e.g., continuous passive motion) in the primary intervention were excluded.

### Search strategy for identifying relevant studies

A McMaster University Health Sciences librarian was consulted during the process of building the search strategy. A literature search was conducted (April 29, 2020) in PubMed, EMBASE, AMED, CINAHL, SPORTDiscus and Cochrane Library using terms that capture exercise, physical therapy, physiotherapy, rehabilitation, adherence, and TKA (Additional file 1: Appendix A). A limiter for RCTs was placed. There was no limiter for date or language. A hand search of references that cited the included full text articles was conducted (June 22, 2020).

### Selection of sources of studies

Initially, eligible studies were uploaded to the referencing software EndNote, and any duplicates were removed. The final list was then uploaded to Covidence, an online screening and data extraction tool. Each screening step (title/abstract and full text) was performed by two independent reviewers (NB, LC) (Additional file 2: Appendix B). If there were any discrepancies between reviewers a third reviewer was consulted (LM). Prior to each screening step, a pilot screening occurred on the first three studies to ensure standardization for selection criteria between each reviewer. Any discrepancy between reviewers in the pilot screening was discussed to identify any concerns and improve reliability.

### Data charting process and items

One pair of investigators independently extracted data from the remaining studies that fit the inclusion criteria (NB, LC). Data was also extracted from any supplemental resources available for each study. Data was independently exported into a data extraction sheet on Microsoft Excel (See Additional file 3: Appendix C). The data extraction table included study design, sample size, descriptive statistics (e.g., age, sex, etc.), exercise intervention type, duration (e.g., 6 weeks, 12 weeks, etc.), outcome (e.g. pain, disability, etc.), and risk of bias as per the Cochrane Risk of Bias Assessment Tool [14].. To assess completeness of exercise reporting we used the Consensus on Exercise Reporting Template (CERT) [15]. The CERT is a 16 item questionnaire that contains specific items related to reporting of exercise interventions [16]. The final score for the CERT is calculated as a total score out of 19. The CERT has been shown to have good inter-rater agreement in trials that included a wide variety of musculoskeletal conditions (e.g., back & neck pain, hip & knee OA) [17]. We assessed the number of items that were reported in each RCT. We considered the reporting to be incomplete if 1 or more items were not reported. To assess exercise adherence, we extracted any definitions of exercise adherence or related terms (e.g., compliance, concordance), and exercise adherence measurements and their outcomes. If there were no explicit mention of any terms that assessed adherence, then general information (e.g., number of sessions attended, completion of exercise diaries) regarding adherence was also included. Outcomes were also collected from the control groups of each included study, as the included studies were parallel RCTs where active interventions were part of the control group. As such, it is important that readers fully understand the components of all interventions included within a study and that the reporting of both interventions allow for replication. Any discrepancies between extractors were assessed by a third-party investigator (LM) if a consensus was not reached.

### Synthesis of results

#### Exercise adherence

Studies were screened electronically for keywords that relate to exercise adherence. Studies were categorized as either reporting exercise adherence (or related terms) or not reporting and presented as a percentage. Studies that reported exercise adherence were assessed for measurement tools used for reporting of exercise adherence (e.g., self-reported patient/practitioner outcomes, accelerometer, etc.), exercise adherence results, and any definitions of exercise adherence. The data from similar outcome measures of exercise adherence were be pooled to assess the level of exercise adherence post-operatively. Definitions of exercise adherence and types of measurements used were presented using summary tables.

#### Intervention reporting

Completeness of intervention reporting was measured as a percentage of intervention completeness, assessing the number of items in the CERT checklist that were included within the study. We reported the median and interquartile range to describe the number of reported items within each RCT. A total score for the CERT, for the intervention and control group of each study, was calculated and reported as mean and standard deviation. Additionally, a description of each item that was not met from the CERT for all exercise interventions in each study was provided in the text analysis. Differences between the number of items met on the CERT between the intervention and control groups were assessed using a paired t-test, with a significance level of 0.05. Of the studies that contained two intervention groups and one control, [18–24] an average of the CERT total score for the intervention was used to conduct the paired t-test. Studies that did not include a control group were excluded from the analysis. Given that the CERT checklist was developed in 2016 and there was no date limit for inclusion of studies in this review, we conducted an independent t-test to assess differences in CERT score prior to and proceeding the publication of the checklist. Cochrane risk of bias was described qualitatively, with studies being labelled with high, low, or unclear risk of bias. STATA IC 15 was used to perform the statistical analysis (t-test), at an alpha level of 0.05 [25].

## Results

### Study selection

**Fig. 1:**
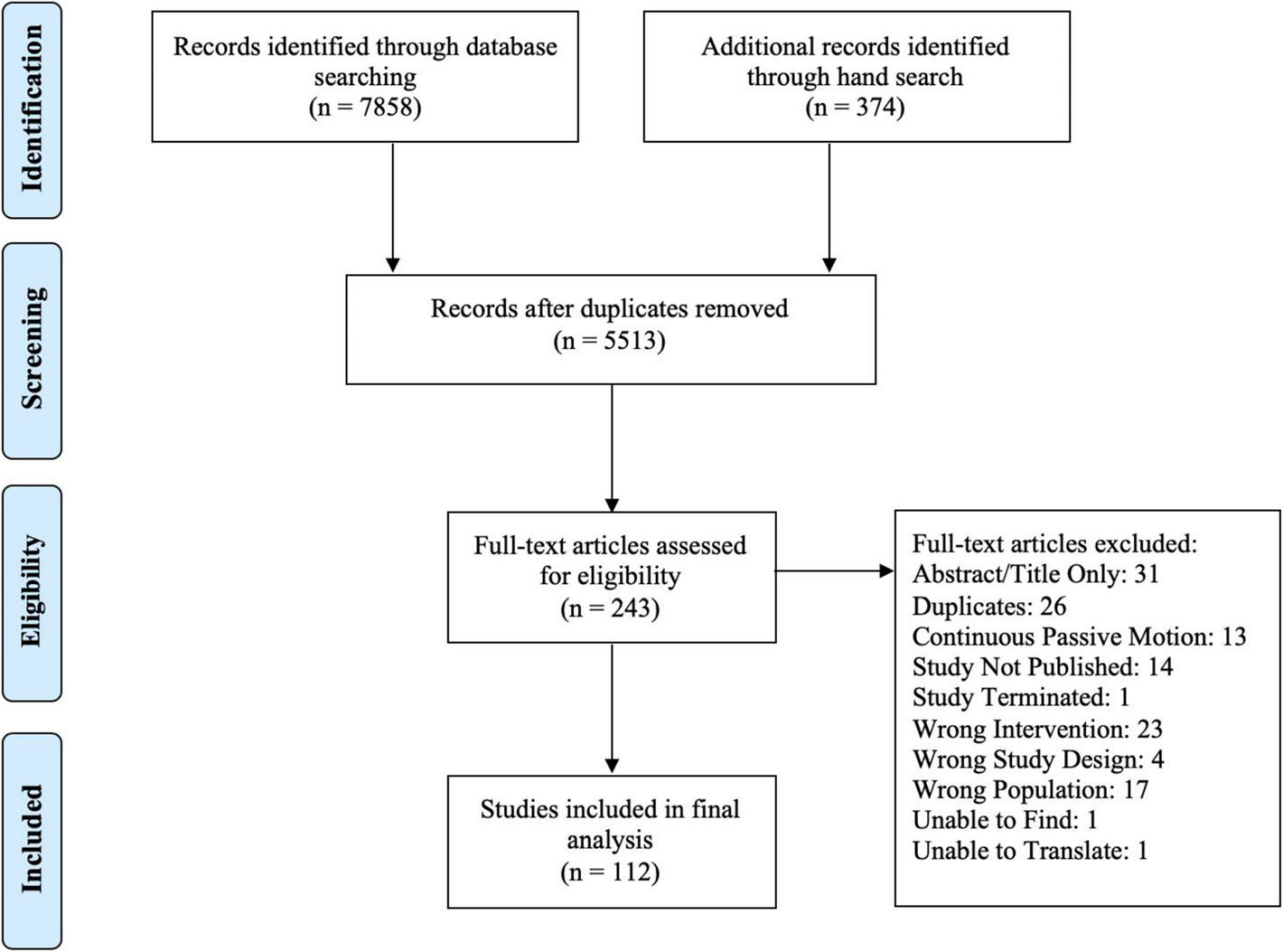
Flow diagram of study selection process

A total of 7858 studies were identified through database searches and a total of 374 through a hand search of reference lists (Fig. 1). Thus, a total of 5513 titles and abstracts were screened after removal of duplicates. Of these studies, 243 citations were considered potentially relevant and were kept for full-text review. A total of 131 citations were excluded because they did not fulfil the inclusion criteria (See Additional file 4: Appendix D for full list of excluded studies with reasons for exclusion). Reasons for exclusion included: 1) Abstract/Title only (e.g., conference proceedings), 2) Duplications, 3) Continuous Passive Motion, 4) Study not yet published, 5) Study terminated, 6) Wrong intervention, study design, or patient population, and 7) Unable to locate/translate study. Finally, 112 RCTs were included in this review [18–24, 26–130].

### General characteristics

The 112 RCTs consisted of a total of 120 intervention groups (more than one treatment arm in some studies) and 112 control groups. These RCTs were conducted in 28 different countries. The top countries included the United States of America (18.8%, *n* = 21), Australia (8.9%, *n* = 10), the United Kingdom (8%, *n* = 9), and Canada/China/Italy/Germany (6.3% respectively, *n* = 7 each).

### Adherence

There were 64 (57%) studies that had mentioned exercise adherence within their study [18, 19, 22–24, 27, 29, 31, 33, 34, 36–38, 42–47, 50, 53, 54, 58, 59, 63, 65–69, 73, 74, 78, 79, 82–84, 86, 90–93, 96–100, 102–104, 107–109, 111, 112, 114, 117, 118, 121, 123, 126, 127, 129, 131]. Of these studies only 41 (65%) reported sufficient information (e.g. measurement used and outcome) to assess exercise adherence [19, 22, 31, 33, 34, 36, 38, 42–45, 53, 58, 66–68, 74, 78, 79, 82, 83, 86, 90–93, 96–98, 102–104, 107–109, 112, 114, 117, 123, 126, 127]. There were 15 studies that had a pre-defined cut-off for exercise adherence (see Table 1). There were 6 studies that stated participants would be excluded or discharged from the study, and thus the analysis, if they failed to meet the adherence cut-offs, which is not in line with the recommended intention-to-treatment analysis [18, 19, 38, 74, 123, 129]. There was one study that evaluated exercise adherence as the primary outcome, but did not provide any conceptual framework around the concept of adherence [91].

**Table 1:** Definitions of exercise adherence in post-operative TKA interventions

Study	Definition of Adherence/Compliance
Akbaba et al. (2016) [18]	“The patients were excluded if they had not completed at least 75% of the exercise programme.”
Buhaglar et al. (2017) [19]	“Adherence for both groups was defined as attendance at no less than 2 and no more than 4 outpatient sessions. Adherence for the inpatient rehabilitation group was further defined as having had a minimum 7 days of inpatient rehabilitation.”
Christiansen et al. (2020) [45]	“We classified adherence as “achieved” for participants who had ≥80% of the weekly steps/day goal recorded by the physical therapist and “not achieved” for those with < 80% of the weekly steps/day goals recorded, which is consistent with the definition of adherence from a pharmacologic perspective.”
Debbi et al. (2019) [47]	“Satisfactory compliance was defined as an average of > 75% of walking protocol.”
Fransen et al. (2017) [58]	“Attended the full program of 16 classes.”
Harmer et al. (2009) [66]	“Attending 8 or more sessions.”
Johnson et al. (2010) [74]	“Subjects were required to complete at least 10 of 12 scheduled therapy sessions. Subjects were allowed to miss up to two sessions, but not in the same week, before being discharged from the study.”
Kelly et al. (2016) [78]	“Goal of 2 sessions per week.”
Kramer et al. (2003) [79]	“Compliance was defined as completion of the home exercises at least 90% of the time.”
Lenguerrand et al. (2019) [82]	“Adherence to the intervention was predefined as attendance at ≥4 sessions.”
Moffet et al. (2015) [98]	“Subjects who participated in all evaluations and attended at least 75% of the intervention sessions.”
Ko et al. (2013) [22]	“Intervention group attended > 9 sessions; the control attended 2 sessions.”
Paxton et al. (2018) [103]	“The adherence rate (in the physical activity feedback group) cutoff of greater than 90% was assessed as the ratio of the number of weeks that the Fitbit wearable sensor and tablet application were used over the total number of weeks. Use of the Fitbit sensor was assessed by noting daily wear time (≥ 12 h representing a valid day). Dose goal cutoff of 80% was assessed as the ratio of the total number of participants achieving their goals over the total number of participants, for each week of the intervention.”
Trudelle-Jackson et al. (2020) [123]	“Participants were considered 100% compliant if they exercised 3 to 4 times per week for 8 weeks (intervention group only). A minimum requirement of 50% compliance with the high-velocity exercise training program was necessary to remain in the study.”
Yousefian et al. (2017) [129]	“Patients not attending 100% of their therapeutic sessions were also excluded from the study.”

Of the 69 reported outcome measures to assess adherence, the number of attended sessions (38%, *n* = 26/69), and patient diary (39%, *n* = 27/69) were the most used (see Table 2). Less commonly used measures of adherence included self-reported patient and clinician questionnaires, activity monitors, computer aided systems (e.g., telehealth app), and duration/intensity of the exercise session. Some studies used multiple measurements when addressing exercise adherence. Adherence was primarily measured during the period of the intervention, which varied from study to study (2–3 day to 8-week intervention periods). Adherence to attended number of sessions ranged from 47 to 100% in the intervention group (*n* = 19), [22, 31, 33, 58, 67, 74, 79, 82, 83, 86, 92, 96–98, 102, 107, 108, 126] and 83 to 100% for the control group (*n* = 10) [22, 33, 74, 79, 83, 86, 98, 107, 108]. Adherence to the assigned exercise sessions as measured using a patient diary ranged from 61 to 110% in the intervention group (*n* = 9), [22, 31, 53, 108, 109, 112, 123, 127] and 65.4 to 85%, in the control group (*n* = 5) [22, 31, 108, 109, 112]. In one study, the authors recommended exercises sessions to be completed 2x/day, and the intervention group completed more exercises than the recommended dose [112]. Only one study reported on the long term adherence to exercises after the intervention phase was completed (49% in the intervention group, and 34% in the control after 12 months) [127].

**Table 2:** Frequency and percentage of commonly measured outcomes of exercise adherence

Exercise Adherence Outcome Measure	Frequency (Percentage)
Activity Monitor	3 (4.35%)
Computer App	3 (4.35%)
Patient Diary	27 (39%)
Number of Attended Sessions	26 (37.68%)
Goal Attainment	1 (1.45%)
Session Duration	4 (5.80%)
Patient Reported Questionnaire	3 (4.35%)
Practitioner Reported Questionnaire	2 (2.90%)

### Completeness of reporting

There were no significant differences in CERT score based on year of publication (e.g., studies published prior to 2016 compared to those published after the development of the questionnaire), both in the intervention (T_110_ = 1.68, *p* < 0.1, mean difference 1.12, CI_95%_ [−0.2, 2.44) and control (T_106_ = 0.47, *p* < 0.6, mean difference 0.34, CI_95%_ [−1.08, 1.75) groups. There were no RCTs that fulfilled all 16 items (total score of 19) of the CERT (See Additional file 5: Appendix E for the completion of the CERT for each included study). There were 18 RCTs that reported at least 14 (74%) items intervention group, [19, 22, 31, 38, 45, 58, 74, 77, 78, 82, 86, 90, 97, 107, 117, 123, 127] and 9 RCTs in the control group [19, 22, 31, 38, 45, 74, 78, 108, 117]. Frequency and percentage of adequately reported CERT items are presented in Table 2. The mean (standard deviation) of adequately reported items in the intervention group was 9.4 (3.5), ranging from 1 to 16 items, which was significantly higher as compared to the control group, 8.0 (3.7), ranging from 1 to17 items, T_107_ = 5.56, *p* < 0.0001, mean difference 1.45, CI_95%_ [0.94, 1.97].

The most reported items in the CERT, in both the intervention and control groups, included mode of delivery (Item 2: Group or individual; Item 3: Supervised or unsupervised), setting (Item 12), and if the intervention was tailored or not (Item 14a), ranging from 80 to 99%. All the other items included in the CERT were not fulfilled on at least 60% of the RCTs. See Table 3 for more detailed information on the reporting of CERT items.

**Table 3:** Frequency and percentage of adequately reported CERT items in the intervention and control groups of post-operative TKA studies

CERT Item	Intervention (*n* = 120)	Control (*n* = 112)
	Frequency (%)	Frequency (%)
Item 1: Exercise Equipment	69 (58%)	32 (31%)
Item 2: Provider Qualifications	10 (8%)	7 (6%)
Item 3: Individual/Group Delivery	114 (95%)	95 (87%)
Item 4: Supervised/Unsupervised	119 (99%)	101(93%)
Item 5: Exercise Adherence	43 (35%)	29 (27%)
Item 6: Motivation	39 (33%)	24 (22%)
Item 7a: Exercise Progression Decision	63 (53%)	38 (35%)
Item 7b: Exercise Progression Description	49 (41%)	29 (28%)
Item 8: Description of Exercise	69 (58%)	46 (43%)
Item 9: Home Program Component	62 (52%)	50 (46%)
Item 10: Non-Exercise Component	63 (52%)	53 (49%)
Item 11: Adverse Events	54 (45%)	49 (45%)
Item 12: Setting of Intervention	110 (92%)	96 (89%)
Item 13: Dosage Total	45 (38%)	32 (30%)
*Sets*	52 (43%)	41 (38%)
*Reps*	50 (42%)	37 (35%)
*Duration*	79 (65%)	57 (53%)
*Intensity*	48 (40%)	29 (27%)
Item 14a: Tailored/Generic	106 (88%)	87 (81%)
Item 14b: Description of Tailoring	9 (23%)	5 (16%)
Item 15: Starting Level of Intervention	50 (42%)	35 (33%)
Item 16a: Intervention Delivered as Planned	40 (33%)	29 (27%)
Item 16b: Fidelity	25 (21%)	23 (21%)

### Cochrane risk of Bias assessment

**Fig. 2:**
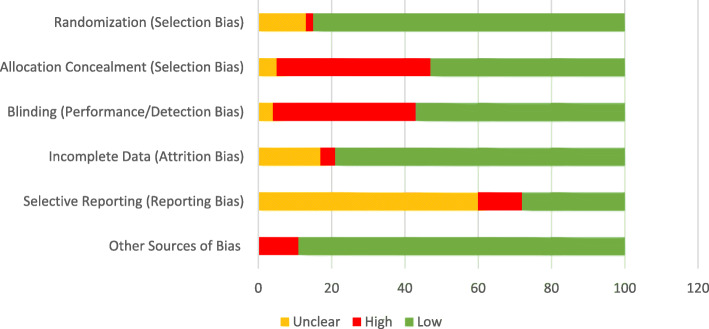
Risk of Bias. The authors’ judgements regarding each domain of the Cochrane Risk of Bias Assessment Tool presented as percentages across all studies

The majority of RCTs included in this review were of poor quality with 16 studies with low risk of bias across all domains, 72 with high risk of bias for one or more of the key domains, and 24 with unclear risk of bias for one or more of the key domains (See Additional file 6: Appendix F for detailed risk of bias assessment). Figure 2 provides a summary of risk of bias judgements for each domain across each all studies.

## Discussion

The RCTs included in this study poorly reported exercise adherence, as well as description of the post-operative TKA rehabilitation intervention. In general, RCTs were of poor quality, with the majority of studies having high or unclear risk of bias across one or more key domains. Similarly, the overall reporting of the CERT items was very poor within the RCTs. While neither intervention was adequately reported, the control group was more likely to be underreported as compared to the intervention group, despite both containing exercise components. These findings are fairly consistent with recent literature which suggests less than one fifth of studies adequately report exercise interventions in other musculoskeletal conditions, [132] educational interventions, [133] as well as in the intensive care unit [134]. Additionally, previous studies have shown that reporting of interventions has improved with time in other health conditions [132]; however, the results of this study show no differences in reporting of interventions over time. This suggests a significant proportion of researchers assessing post-operative TKA interventions have not adapted the newer recommendations for reporting of interventions.

Of the studies that mentioned exercise adherence, very few provided a definition of adherence in the context of their study. A compounding problem, however, is that some that defined adherence included pre-defined cut-offs, [18, 19, 38, 74, 123, 129] in which participants would be excluded from the study and analysis if they did not meet them. These cut-offs were different in each study and were not based on any scientific rationale for assessing adherence. As such, intent-to-treat analysis was not used, which may overestimate the effectiveness of the intervention. Authors should properly define exercise adherence and treat adherence as a continuous measure such that statistical procedures may address the issue of nonadherence.

One interesting, but not surprising, finding of this review is that the terms adherence and compliance were used interchangeably. Compliance and adherence, however, are inherently different constructs. Compliance refers to “the extent to which the patient’s behaviour matches the prescriber’s recommendations.” [135] While adherence, according to the WHO, refers to “the extent to which a person’s behaviour, taking medication, following a diet, and/or executing lifestyle changes, corresponds with agreed recommendations from a health care provider.” [135] In this case, adherence refers to a process in which the agreed upon treatment is discussed with the patient. In this way, the patient is not the only person responsible for non-adherence but places an onus on the clinician as well. A recent systematic review of the validity, reliability, and acceptability of exercise adherence measures provided an extension to the WHO definition: “The extent to which individuals undertake prescribed behaviour accurately and at the agreed frequency, intensity and duration.” [9] It is clear, however, that there is no agreed upon definition of exercise adherence and the most commonly used definition by the WHO lacks specific concepts (e.g., frequency, intensity, accuracy, etc.), which are important in adherence to exercise [136].

Outcome measures used to assess adherence varied between studies including subjective (e.g., diary, self-reported patient/practitioner questionnaire) and objective measures (e.g., accelerometers, number of attended sessions). Exercise adherence was commonly measured as the number of supervised exercise sessions and self-reported exercise diary. These methods, however, have been reported to have poor validity and reliability [9]. The inconsistency in measurements used to assess adherence limits the ability to draw conclusions regarding accuracy and compare between studies [16]. These findings are consistent with previous literature that suggests lack of reporting of adherence in exercise interventions [137]. Lack of proper reporting of adherence may lead to underestimation of treatment effects, in that, participants may not be improving after an exercise intervention due to lack of adherence, rather than an ineffective intervention, and will limit clinical application and proper understanding of the burden of the intervention on the patient. Future studies should aim to validate existing measures of exercise adherence that can be used to accurately assess this construct within a clinical trial. It is recommended that authors use valid and reliable outcome measures of exercise adherence across all study treatment arms.

Poor reporting for post-operative TKA exercise interventions is particularly concerning as it limits reproducibility of research, but more importantly, it limits translation into clinical practice. The majority of RCTs included in this study failed to report more than 70% of the CERT items in both the intervention and control group. This is consistent with recent literature that assessed reporting interventions for LBP [132]. In particular, description of the exercises, dosage, progression of exercise, adverse events, content of home program/non-exercise components, and fidelity were not well reported. These items may directly relate to exercise adherence, and thereby, influence uptake of exercise in the long-term. Based on the results of this study, it is unclear if authors did not include these vital components within their interventions, or simply failed to report them. Future studies should aim to report all recommended items within an intervention reporting checklist and provide rationale if items were not included within their intervention.

Without adequate information of how interventions are developed or implemented, studies recommending rehabilitation for post-operative TKA should be interpreted with caution. Inadequate reporting of specific rehabilitation procedures may lead to improper clinical application, leading to potential harm [132]. These items are particularly important in intervention reporting to reduce bias in implementation, as well as standardization for future studies or clinical replications. Poor reporting can be, in part, attributed to the complexity of rehabilitation interventions [138] leading to difficulty of standardization [139].

### Limitations and strengths

This study had several limitations, first publication bias may influence adherence results as studies that have poor adherence are generally less likely to be published [140]. The purpose of this study was to evaluate the reporting of intervention within primary research articles, and as such, protocol studies were excluded from this review. Some research articles describe their interventions within their published protocols, and this may have been missed in the overall reporting of this study. Since the purpose of this scoping review was to assess the completeness of intervention reporting, there was no considerations of effectiveness of each intervention. The strengths of this study relate to the use of a framework that provides transparency and replicability. Additionally, we used multiple databases that allowed us to capture most studies that assessed post-op TKA rehabilitation. This study also looked at both the control and intervention groups separately using a validated reporting tool as well as the validated Cochrane Risk of Bias assessment tool, which ensures replicability as well as a comprehensive analysis of the current literature in post-op TKA rehabilitation.

## Conclusion

In conclusion, exercise adherence is poorly reported within studies of post-operative TKA rehabilitation. The majority of studies measured exercise adherence through the use of patient diaries, or number of attended session. Further, very few studies provided a defintition of adherence. A such, future studies should aim to identify accurate and reliable measures of adherence, and authors should include an exercise reporting tool when planning/implementing exercise interventions as well as provide a clear definition of exercise adherence. In general, the completeness of reporting components of both the intervention and control groups was very poor. Proper reporting will allow for smoother translation of research into clinical practice as well as better quality and reproducibility of research.

## Supplementary Information

**Additional file 1: Appendix A.** Search Strategy Example.

**Additional file 2: Appendix B.** Inclusion Criteria Table.

**Additional file 3: Appendix C.** Data Extraction Table.

**Additional file 4: Appendix D.** List of Excluded Studies.

**Additional file 5: Appendix E.** CERT Analysis.

**Additional file 6: Appendix F.** Cochrane Risk of Bias Assessment.

## Data Availability

The datasets used and/or analysed during the current study may be extracted from the original articles or will be made available from the corresponding author on upon request.

## Abbreviations

TKA: Total knee arthroplasty
RCT: Randomized Controlled Trial
CERT: Consensus on Exercise Reporting Template
