# Supplementary material for: Reporting of post-operative rehabilitation interventions for Total knee arthroplasty: a scoping review
Source: BMC Musculoskelet Disord. 2021 Jun 30;22:602. doi: 10.1186/s12891-021-04460-w (PMC8247251; doi:10.1186/s12891-021-04460-w)
Supplement: Supplementary file 1 — Additional file 1: Appendix A. Search Strategy Example. [file 12891_2021_4460_MOESM1_ESM.docx]

## Appendix A – Search Strategy Example

**EMBASE**

1

exp exercise/

2

exp physiotherapy/

3

exp rehabilitation/

4

exp rehabilitation care/

5

exp kinesiotherapy/

6

exercise*.ab,ti,kw.

7

physiotherap*.ab,ti,kw.

8

physical therap*.ab,ti,kw.

9

rehabilitation.ab,ti,kw.

10

rh.fs.

11

1 or 2 or 3 or 4 or 5 or 6 or 7 or 8 or 9 or 10

12

exp knee/

13

knee.ab,ti,kw.

14

12 or 13

15

exp arthroplasty/

16

exp total knee arthroplasty/

17

exp knee replacement/

18

14 and 15

19

exp knee osteoarthritis/su [Surgery]

20

knee replacement*.ab,ti,kw.

21

knee arthroplast*.ab,ti,kw.

22

(knee adj3 (replacement* or arthroplast*)).ti,ab,kw.

23

exp postoperative care/

24

14 and 23

25

15 or 16 or 17 or 18 or 19 or 20 or 21 or 22 or 24

26

exp clinical trial/

27

exp controlled study/

28

controlled trial*.ti,ab,kw.

29

clinical trial*.ti,ab,kw.

30

single blind*.ti,ab,kw.

31

double blind*.ti,ab,kw.

32

rct.ti,ab,kw.

33

randomi?ed controlled trial*.ti,ab,kw.

34

random.ti,ab,kw.

35

26 or 27 or 28 or 29 or 30 or 31 or 32 or 33 or 34
