# Supplementary material for: Reporting of post-operative rehabilitation interventions for Total knee arthroplasty: a scoping review
Source: BMC Musculoskelet Disord. 2021 Jun 30;22:602. doi: 10.1186/s12891-021-04460-w (PMC8247251; doi:10.1186/s12891-021-04460-w)
Supplement: Supplementary file 2 — Additional file 2: Appendix B. Inclusion Criteria Table. [file 12891_2021_4460_MOESM2_ESM.docx]

**Appendix B – Inclusion Criteria Table**

| Table 1. Inclusion criteria for title, abstract and full-text screening | |
| --- | --- |
| Reviewer:  First author:  Year: | |
| Participants: Adults (18 years or older), patients that underwent bilateral or unilateral TKA |  |
| Type of Study: Randomized Control Trial |  |
| Language: Any |  |
| Intervention: Exercise-based intervention (Adult population (18 years or older)) that includes physical exercises such as strengthening, flexibility, and/or aerobic activities. |  |
