# Supplementary material for: Reporting of post-operative rehabilitation interventions for Total knee arthroplasty: a scoping review
Source: BMC Musculoskelet Disord. 2021 Jun 30;22:602. doi: 10.1186/s12891-021-04460-w (PMC8247251; doi:10.1186/s12891-021-04460-w)
Supplement: Supplementary file 3 — Additional file 3: Appendix C. Data Extraction Table. [file 12891_2021_4460_MOESM3_ESM.docx]

## Appendix C – Data Extraction Table

| **Item** |  |
| --- | --- |
| **Author** |  |
| **Title** |  |
| **Year** |  |
| **Journal** |  |
| **Funding** |  |
| **Location** |  |
| **Randomization sequence generation** |  |
| **Allocation concealment** |  |
| **Blinding** |  |
| **Incomplete Outcome Data Address** |  |
| **Lack of Selective Reporting** |  |
| **Lack of Other Sources of Bias** |  |
| **Evaluation of Risk of Bias** |  |
| **Type of Intervention** |  |
| **Type of Exercise Equipment** |  |
| **Qualifications** |  |
| **Individual/Group** |  |
| **Supervised/Unsupervised** |  |
| **Decision for Progression** |  |
| **Motivation Strategies** |  |
| **Exercise Description (e.g. photos, etc.)** |  |
| **Content of Home Component** |  |
| **Non-exercise components** |  |
| **Adverse event management** |  |
| **Setting of Exercises** |  |
| **Sets** |  |
| **Repetitions** |  |
| **Duration** |  |
| **Intensity** |  |
| **Generic or tailored to individual** |  |
| **Decision for starting level** |  |
| **Intervention Delivered as Planned** |  |
| **Control** |  |
| **Type of Exercise Equipment** |  |
| **Qualifications** |  |
| **Individual/Group** |  |
| **Supervised/Unsupervised** |  |
| **Decision for Progression** |  |
| **Motivation Strategies** |  |
| **Description of exercise (e.g. photos, etc)** |  |
| **Content of Home Component** |  |
| **Non-exercise components** |  |
| **Adverse event management** |  |
| **Definition of Adherence** |  |
| **Adherence Measurement Used** |  |
| **Adherence Outcome Results** |  |
| **Adherence/Fidelity** |  |
