# Supplementary material for: Reporting of post-operative rehabilitation interventions for Total knee arthroplasty: a scoping review
Source: BMC Musculoskelet Disord. 2021 Jun 30;22:602. doi: 10.1186/s12891-021-04460-w (PMC8247251; doi:10.1186/s12891-021-04460-w)
Supplement: Supplementary file 4 — Additional file 4: Appendix D. List of Excluded Studies. [file 12891_2021_4460_MOESM4_ESM.docx]

## Appendix D – List of Excluded Studies

| **Reference** | **Reason for Exclusion** |
| --- | --- |
| [Actrn (2013) The effect of group work-circuit and individualised physiotherapy treatments for orthopaedic patients: a randomised control trial. http://www.who.int/trialsearch/Trial2.aspx?TrialID=ACTRN12613001196730](http://www.who.int/trialsearch/Trial2.aspx?TrialID=ACTRN12613001196730) | Duplicate |
| [Actrn (2016) The STEP study: a Pragmatic, randomized, controlled trial comparing two post operative management pathways in knee or hip replacement surgery. http://www.who.int/trialsearch/Trial2.aspx?TrialID=ACTRN12616000696493](http://www.who.int/trialsearch/Trial2.aspx?TrialID=ACTRN12616000696493) | Study not published yet |
| [Actrn (2017) A pedaling-based three exercise protocol compared to a non-pedaling ten exercise protocol for immediate post-operative rehabilitation after total knee replacement in a randomized controlled trial. http://www.who.int/trialsearch/Trial2.aspx?TrialID=ACTRN12617000647336](http://www.who.int/trialsearch/Trial2.aspx?TrialID=ACTRN12617000647336) | Study not published yet |
| [Actrn (2019) Face-to-face physiotherapy compared to a supported home exercise program for the management of musculoskeletal conditions: the REFORM trial. http://www.who.int/trialsearch/Trial2.aspx?TrialID=ACTRN12619000065190](http://www.who.int/trialsearch/Trial2.aspx?TrialID=ACTRN12619000065190) | Study not published yet |
| Alnahdi AH, Zeni JA, Snyder-Mackler L (2012) The effect of progressive strengthening programs on function and gait mechanics after unilateral total knee arthroplasty: a randomized clinical trial. Osteoarthritis and Cartilage. 20:S104‐S105 | Abstract/Title Only |
| Alobaidi A, Mahmood A (2018) Effect of continuous passive motion (CPM) exercises on post total knee arthroplasty (TKA) rehabilitation. Osteoporos Int 29 (1 Supplement 1):S162-S163 | Abstract/Title Only |
| Andersen HH (2018) Technological assisted rehabilitation following total knee joint replacement. a randomised controlled non-inferiority trial. Ann Rheum Dis 77:1823‐1824 | Abstract/Title Only |
| Antall GF, Kresevic D (2004) The use of guided imagery to manage pain in an elderly orthopaedic population. Orthopaedic nursing / National Association of Orthopaedic Nurses 23:335-340 | Wrong Intervention |
| Antony-Leo AP, Arun-Maiya G, Mohan-Kumar M, Vijayaraghavan PV (2019) Structured total knee replacement rehabilitation programme and quality of life following two different surgical approaches - A randomised controlled trial. Malaysian Orthopaedic Journal 13:20‐27 | Wrong Intervention |
| Atabaki S, Farahani MA, Haghani S (2019) Effect of rehabilitation education on pain, knee stiffness and performance difficulty in patients undergoing knee replacement surgery: a randomized clinical trial. Journal of Acute Disease 8:233‐238 | Wrong Intervention |
| Bade MJ, Christensen JC, Zeni JA, Christiansen CL, Dayton MR, Forster JE, et al. (2020) Movement pattern biofeedback training after total knee arthroplasty: Randomized clinical trial protocol. Contemp Clin Trials 91 (no pagination): | Wrong Study Design |
| Bandholm T, Kehlet H (2012) Physiotherapy Exercise After Fast-Track Total Hip and Knee Arthroplasty: Time for Reconsideration?...Liebs TR, Herzberg W, Ruther W, Haasters J, Russlies M, Hassenpflug J. Multicenter randomized controlled trial comparing early versus late aquatic therapy after total hip or knee arthroplasty. Arch Phys Med Rehabil 2012;93:192-9. Arch Phys Med Rehabil 93:1292-1294 | Wrong Study Design |
| Beaupre L, Davies D, Jones C, Cinats J (2001) Exercise combined with continuous passive motion or slider board therapy compared with exercise only: a randomized controlled trial of patients following total knee arthroplasty. Phys Ther 81:1029-1037. | Includes Passive Motion |
| Bellelli G, Buccino G, Garrah M, Padovani A, Trabucchi M (2009) Action observation treatment in the rehabilitation of post-surgical orthopaedic patients: a randomised controlled trial. Eur J Neurol 16:308‐ | Abstract/Title Only |
| Bethge M, Bartel S, Streibelt M, Lassahn C, Thren K (2011) Improved outcome quality following total knee and hip arthroplasty in an integrated care setting: results of a controlled study]. Rehabilitation (Stuttg) 50:86-93 | Wrong Intervention |
| Bettger J, Green CL, Holmes DN, Chokshi A, Mather RC, III, Hoch BT, et al. (2020) Effects of Virtual Exercise Rehabilitation In-Home Therapy Compared with Traditional Care After Total Knee Arthroplasty VERITAS, a Randomized Controlled Trial. Journal of Bone and Joint Surgery-American Volume 102:101-109 | Duplicate |
| Boissy P, Tousignant M, Moffet H, Nadeau S, Briere S, Merette C, et al. (2016) Conditions of Use, Reliability, and Quality of Audio/Video-Mediated Communications During In-Home Rehabilitation Teletreatment for Postknee Arthroplasty. Telemedicine journal and e-health : the official journal of the American Telemedicine Association 22:637-649 | Wrong Study Design |
| Borgwardt L, Zerahn B, Bliddal H, Christiansen C, Sylvest J, Borgwardt A (2009) Similar clinical outcome after unicompartmental knee arthroplasty using a conventional or accelerated care program: a randomized, controlled study of 40 patients. Acta Orthop 80:334‐337 | Wrong Intervention |
| Browne JA (2019) After Unilateral Total Knee Arthroplasty, Unsupervised Home Exercise Programs Were Noninferior to Outpatient Physiotherapy Services for Increasing Passive Flexion. Journal of Bone and Joint Surgery - American Volume 101:2063 | Wrong Study Design |
| Buhagiar MA, Naylor JM, Harris IA, Xuan W, Kohler F, Wright R, et al. (2017) Effect of Inpatient Rehabilitation vs a Monitored Home-Based Program on Mobility in Patients With Total Knee Arthroplasty The HIHO Randomized Clinical Trial. Jama-Journal of the American Medical Association 317:1037-1046 | Duplicate |
| Cawthorne D, March L, Parker D, Coolican M, Negus J (2015) TKR-power-patient outcomes using wii enhanced rehabilitation after a total knee replacement. Physiotherapy (united kingdom). 101:eS204‐eS205 | Abstract/Title Only |
| Chen G, Gu RX, Xu DD (2012) The application of electroacupuncture to postoperative rehabilitation of total knee replacement. Zhongguo zhen jiu [Chinese acupuncture & moxibustion] 32:309‐312 | Includes Passive Motion |
| [Chi CI (2016) Effects of joint mobilization techniques in primary total knee arthroplasty: a single-blind randomized controlled trial. http://www.who.int/trialsearch/Trial2.aspx?TrialID=ChiCTR-IOR-16009192](http://www.who.int/trialsearch/Trial2.aspx?TrialID=ChiCTR-IOR-16009192) | Wrong Study Design |
| Chow TPY, Ng GYF (2010) Active, passive and proprioceptive neuromuscular facilitation stretching are comparable in improving the knee flexion range in people with total knee replacement: a randomized controlled trial. Clin Rehabil 24:911-918 | Includes Passive Motion |
| Christiansen M, Thoma L, Master H, Mathews D, Schmitt L, Ziegler M, et al. (2018) 1-year outcomes from a novel physical therapist-administered physical activity intervention after total knee replacement: a pilot study. Arthritis and Rheumatology 70:439‐440 | Abstract/Title Only |
| Christiansen MB, Thoma LM, Master H, Mathews D, Schmitt LA, White DK (2018) Preliminary findings of a novel physical therapist administered physical activity intervention after total knee replacement. Osteoarthritis Cartilage 26 (Supplement 1):S334 | Abstract/Title Only |
| Christiansen MB, Thoma LM, Master H, Voinier D, Schmitt LA, Ziegler ML, et al. (2019) The feasibility and preliminary outcomes of a physical therapist-administered physical activity intervention after total knee replacement. Arthritis Care Res (Hoboken);10.1002/acr.23882 | Abstract/Title Only |
| Chughtai M, Newman JM, Sultan AA, Khlopas A, Navarro SM, Bhave A, et al. (2018) The Role of Virtual Rehabilitation in Total Knee and Hip Arthroplasty. Surg Technol Int 32:299-305 | Wrong Study Design |
| Codine P, Dellemme Y, Denis-Laroque F, Herisson C (2004) The use of low velocity submaximal eccentric contractions of the hamstring for recovery of full extension after total knee replacement: a randomized controlled study. Isokinet Exerc Sci 12:215-218 | Includes Passive Motion |
| Cooke M, Walker R, Aitken L, Freeman A, Pavey S, Cantrill R (2016) Pre-operative self-efficacy education vs. usual care for patients undergoing joint replacement surgery: A pilot randomised controlled trial. Scand J Caring Sci 30:74-82. | Wrong Study Design |
| [Ctri (2018) EFFECTIVENESS OF TELEPHONICALLY REINFORCED HOME EXERCISE PROGRAM FOLLOWING TOTAL KNEE REPLACEMENT: - AN ASSESSOR BLINDED RANDOMIZED CONTROLLED TRIAL. http://www.who.int/trialsearch/Trial2.aspx?TrialID=CTRI/2018/09/015578](http://www.who.int/trialsearch/Trial2.aspx?TrialID=CTRI/2018/09/015578) | Study not published yet |
| Dan M, Boca I-C (2013) BENEFICIILE KINETOTERAPIEI ÎN PROGRAMUL DE RECUPERARE FUNCŢIONALĂ DUPĂ ARTROPLASTIA TOTALĂ DE GENUNCHI. Romanian Journal of Physical Therapy / Revista Romana de Kinetoterapie 19:23-28 | Unable to Find |
| Dan M, Boca I-C (2013) PERKS OF PHYSICAL THERAPY DUE TO A COMPLEX REHABILITATION PROGRAM AFTER TOTAL KNEE ARTHROPLASTY. Romanian Journal of Physical Therapy / Revista Romana de Kinetoterapie 19:23-28 | Wrong Study Design |
| Debbi EM, Bernfeld B, Soudry M, Salai M, Laufer Y, Herman A, et al. (2014) A biomechanical therapy program for patients after total knee arthroplasty - A randomized controlled trial (preliminary results). Osteoarthritis Cartilage 22:S82 | Abstract/Title Only |
| Doiron-Cadrin P, Kairy D, Vendittoli PA, Lowry V, Poitras S, Desmeules F (2020) Feasibility and preliminary effects of a tele-prehabilitation program and an in-person prehablitation program compared to usual care for total hip or knee arthroplasty candidates: a pilot randomized controlled trial. Disabil Rehabil 42:989-998 | Wrong Intervention |
| Dong F, Li M, Liu J (2017) Effect of department of orthopedics rehabilitation integrated mode on knee joint pain, function and quality of life in total knee arthroplasty. Biomedical Research (India) 28:9534-9537 | Includes Passive Motion |
| Dowsey MM, Kilgour ML, Santamaria NM, Choong PF (1999) Clinical pathways in hip and knee arthroplasty: a prospective randomised controlled study. Med J Aust 170:59‐62 | Wrong Intervention |
| Eberle T, Rossi MD, Roche M, Blake R, Burwell B, Elbaum L, et al. (2008) Utilization of an exercise protocol with the distal segment fixed following primary unilateral knee replacement: a descriptive analysis...2008 Combined Sections Meeting...Nashville, Tennessee, February 6-9, 2008. J Orthop Sports Phys Ther 38:A45-46 | Abstract/Title Only |
| Fillingham YA, Darrith B, Lonner JH, Culvern C, Crizer M, Della Valle CJ (2018) Formal Physical Therapy May Not Be Necessary After Unicompartmental Knee Arthroplasty: A Randomized Clinical Trial. J Arthroplasty 33:S93-S99.e93 | Wrong Patient Population |
| Fukushima H, Takahashi S, Miyahara H (2009) Knee Joint Mobility Differs with Start Day of Range of Motion Exercise after Total Knee Arthroplasty. Rigakuryoho Kagaku 24:391-395 | Includes Passive Motion |
| Fung V, Ho A, Shaffer J, Gomez M (2010) Poster 106: The Utilization of Nintendo Wii Fit in the Rehabilitation of Outpatients Following Total Knee Replacements: Preliminary Results of a Randomized Controlled Trial. Arch Phys Med Rehabil 91:e37-e37 | Abstract/Title Only |
| Fung V, Ho A, Shaffer J, Gomez M (2010) The utilization of nintendo wii fit in the rehabilitation of outpatients following total knee replacements: preliminary results of a randomized controlled trial. Arch Phys Med Rehabil 91:e37‐ | Abstract/Title Only |
| Fung V, Shaffer J, Chung E, Ho A, Gomez M (2011) The utilization of nintendo wii fittm in the rehabilitation of outpatients following total knee replacements-a randomized controlled trial. Physiotherapy (United Kingdom) 97:eS419‐ | Abstract/Title Only |
| Ganz SB, Ranawat CS (2004) Efficacy of formal knee flexion exercises on the achievement of functional milestones following total knee arthroplasty. Topics in Geriatric Rehabilitation 20:311-311 | Abstract/Title Only |
| Gao Zy, Wu Jx, Lin Wl, Tuo M, Zhu Qx, Wei L, et al. (2011) Comprehensive rehabilitation following total knee arthroplasty: A randomized controlled trial. Journal of Clinical Rehabilitative Tissue Engineering Research 15:7275-7278 | Includes Passive Motion |
| Gooch K, Marshall DA, Faris PD, Khong H, Wasylak T, Pearce T, et al. (2012) Comparative effectiveness of alternative clinical pathways for primary hip and knee joint replacement patients: a pragmatic randomized, controlled trial. Osteoarthritis Cartilage 20:1086-1094 | Wrong Intervention |
| Guney H, Vardar Yagli N, Caglar O, Yuksel I (2014) Effects of relaxation techniques in total knee arthroplasty patients during their hospital stay. Ann Rheum Dis 73: | Abstract/Title Only |
| Hadley C, McGrath M, Prodoehl JP, Cohen SB, Emper WD, Hammoud S, et al. (2019) Comparison of Traditional Physical Therapy to Internet-Based Physical Therapy after Knee Arthroscopy: A Prospective Randomized Controlled Trial Comparing Patient Outcomes and Satisfaction...American Orthopaedic Society for Sports Medicine Annual Meeting, July 11-14, 2019, Boston, Massachusetts. Orthopaedic Journal of Sports Medicine 7:1-2 | Wrong Patient Population |
| Hamilton D, Beard D, Barker K, MacFarlane G, Murray G, Simpson H (2019) Targeting physiotherapy to patients at risk of poor outcomes following total knee arthroplasty: the TRIO randomised controlled trial...The Chartered Society of Physiotherapy UK Conference 2018, Birmingham, UK, 19-20 October 2018. Physiotherapy 105:e160-e161 | Abstract/Title Only |
| Hasubhai PZ, D BD, A TR (2017) Effectiveness of Conventional Physiotherapy along with Continuous Passive Motion after Total Knee Arthroplasty. Indian Journal of Physiotherapy & Occupational Therapy 11:195-200 | Includes Passive Motion |
| Hershko E, Tauber C, Carmeli E (2008) Biofeedback versus physiotherapy in patients with partial weight-bearing. Am J Orthop (Belle Mead NJ) 37:E92-96 | Wrong Patient Population |
| Hsu WH, Hsu WB, Shen WJ, Lin ZR, Chang SH, Hsu RWW (2019) Twenty-four-week hospital-based progressive resistance training on functional recovery in female patients post total knee arthroplasty. Knee 26:729-736 | Wrong Study Design |
| Huang P, He J, Zhang YM (2017) [The mobile application of patient management in education and follow-up for patients following total knee arthroplasty]. Chung-Hua i Hsueh Tsa Chih [Chinese Medical Journal] 97:1592-1595 | Wrong Study Design |
| Ibanez AE (2008) Can a functional rehabilitation program improve the outcome and quality of life of patients with total knee arthroplasty?. [Spanish]. FMC Formacion Medica Continuada en Atencion Primaria 15:338 | Wrong Study Design |
| [Isrctn (2004) Comparison of post-discharge physiotherapy versus usual care following total knee replacement: a randomised clinical trial. http://www.who.int/trialsearch/Trial2.aspx?TrialID=ISRCTN07624314](http://www.who.int/trialsearch/Trial2.aspx?TrialID=ISRCTN07624314) | Study not published yet |
| [Isrctn (2004) Pilot randomised controlled trial of an early rehabilitation class versus outpatient physiotherapy following discharge from hospital after primary knee arthroplasty. http://www.who.int/trialsearch/Trial2.aspx?TrialID=ISRCTN40417508](http://www.who.int/trialsearch/Trial2.aspx?TrialID=ISRCTN40417508) | Duplicate |
| [Isrctn (2016) Activity orientated rehabilitation following knee arthroplasty: feasibility study. http://www.who.int/trialsearch/Trial2.aspx?TrialID=ISRCTN13579789](http://www.who.int/trialsearch/Trial2.aspx?TrialID=ISRCTN13579789) | Study not published yet |
| [Isrctn (2018) PEP-TALK: a study investigating whether having group discussions in addition to physiotherapy improves the amount of physical activity following hip and knee replacement. http://www.who.int/trialsearch/Trial2.aspx?TrialID=ISRCTN29770908](http://www.who.int/trialsearch/Trial2.aspx?TrialID=ISRCTN29770908) | Duplicate |
| [Isrctn (2018) The effect of the Mulligan mobilization with movement approach following knee replacement surgery. http://www.who.int/trialsearch/Trial2.aspx?TrialID=ISRCTN13028992](http://www.who.int/trialsearch/Trial2.aspx?TrialID=ISRCTN13028992) | Study not published yet |
| Jayabalan P, Almeida GJ, Wan H, Sowa GA, Piva SR (2014) THE INVESTIGATION OF CANDIDATE BIOMARKERS TO ASSESS THE EFFICACY OF A NOVEL REHABILITATION REGIMEN FOLLOWING TOTAL KNEE ARTHROPLASTY: A PILOT STUDY. Am J Phys Med Rehabil a72-a73 | Abstract/Title Only |
| Jogi P, Zecevic A, Overend TJ, Spaulding SJ, Kramer JF (2016) Force-plate analyses of balance following a balance exercise program during acute post-operative phase in individuals with total hip and knee arthroplasty: A randomized clinical trial. SAGE Open Medicine 4: | Duplicate |
| Jørgensen PB, Bogh SB, Kierkegaard S, Sørensen H, Odgaard A, Søballe K, et al. (2017) The efficacy of early initiated, supervised, progressive resistance training compared to unsupervised, home-based exercise after unicompartmental knee arthroplasty: a single-blinded randomized controlled trial. Clin Rehabil 31:61-70 | Wrong Patient Population |
| Kauppila AM, Sintonen H, Aronen P, Ohtonen P, Kyllonen E, Arokoski JPA (2011) Economic evaluation of multidisciplinary rehabilitation after primary total knee arthroplasty based on a randomized controlled trial. Arthritis Care Res 63:335-341 | Duplicate |
| Kierkegaard S, Jorgensen PB, Soballe K, Mechlenburg I (2015) Pelvic movements are restored to reference values during stair climbing but not during stepping one year after uni-compartmental knee arthroplasty-a secondary analysis of a randomized controlled trial. Osteoarthritis Cartilage 23:A111-A112 | Abstract/Title Only |
| Ko V, Naylor J, Harris I, Crosbie J, Yeo A, Mittal R (2013) One-to-One Therapy Is Not Superior to Group or Home-Based Therapy After Total Knee Arthroplasty A Randomized, Superiority Trial. Journal of Bone and Joint Surgery-American Volume 95A:1942-1949 | Abstract/Title Only |
| Ko VWM, Naylor JM, Harris IA, Yeo AET, Crosbie J (2011) Is centre-based rehabilitation superior to home-based rehabilitation after knee replacement? A single-blind, randomised controlled trial. Arthritis Rheum 63:4043‐ | Duplicate |
| Kösters A, Pötzelsberger B, Dela F, Dorn U, Hofstaedter T, Fink C, et al. (2015) A lpine S kiing W ith total knee ArthroPlasty (ASWAP): study design and intervention. Scand J Med Sci Sports 25:3-9 | Wrong Study Design |
| Kosters A, Rieder F, Wiesinger HP, Dorn U, Hofstaedter T, Fink C, et al. (2015) Alpine Skiing With total knee ArthroPlasty (ASWAP): effect on tendon properties. Scand J Med Sci Sports 25:67-73 | Wrong Study Design |
| Kotani N, Morishita T, Saita K, Kamada S, Maeyama A, Abe H, et al. (2019) Feasibility of supplemental robot-assisted knee flexion exercise following total knee arthroplasty. Journal of back and musculoskeletal rehabilitation. 10: | Includes Passive Motion |
| Kristensen MS, Jorgensen PB, Bogh SB, Kierkegaard S, Mechlenburg I, Dalgas U (2018) Acute and chronic effects of early progressive resistance training on knee pain and knee joint effusion after unicompartmental knee arthroplasty. Acta Orthop Belg 84:262-268 | Duplicate |
| Kuiken TA, Amir H, Scheidt RA (2004) Computerized biofeedback knee goniometer: Acceptance and effect on exercise behavior in post-total knee arthroplasty rehabilitation. Arch Phys Med Rehabil 85:1026-1030 | Wrong Study Design |
| Labraca NS, Castro-Sánchez AM, Matántilde, rdn-Penarrocha GA, Arroyo-Morales M, Moreno-Lorenzo C (2012) Starting Rehabilitation within 24 Hours After Total Knee Arthroplasty Was Better Than Delaying to within 48 to 72 Hours. Journal of Bone & Joint Surgery, American Volume 94:366-366 | Abstract/Title Only |
| Labraca NS, Castro-Sanchez AM, Mataran-Penarrocha GA, Arroyo-Morales M, Sanchez-Joya MM, Moreno-Lorenzo C (2011) Benefits of starting rehabilitation within 24 hours of primary total knee arthroplasty: randomized clinical trial. Clin Rehabil 25:557-566 | Includes Passive Motion |
| Larsen K, Hansen TB, Thomsen PB, Christiansen T, Søballe K, Larsen K, et al. (2009) Cost-effectiveness of accelerated perioperative care and rehabilitation after total hip and knee arthroplasty. Journal of Bone & Joint Surgery, American Volume 91:761-772 | Wrong Study Design |
| Larsen K, Sørensen OG, Hansen TB, Thomsen PB, Søballe K (2008) Accelerated perioperative care and rehabilitation intervention for hip and knee replacement is effective: a randomized clinical trial involving 87 patients with 3 months of follow-up. Acta Orthop 79:149-159 | Wrong Intervention |
| Lee YK, Kim BR (2016) Effects of an early eccentrically based rehabilitation after total knee arth-roplasty. Osteoporosis international. Conference: world congress on osteoporosis, osteoarthritis and musculoskeletal diseases, WCO-IOF-ESCEO 2016. Malaga spain. Conference start: 20160414. Conference end: 20160417. Conference publication: (var.pagings) 27:S88 | Abstract/Title Only |
| Li J, Wu T, Xu Z, Gu X (2014) A pilot study of post-total knee replacement gait rehabilitation using lower limbs robot-assisted training system. European journal of orthopaedic surgery & traumatology : orthopedie traumatologie 24:203‐208 | Wrong Intervention |
| Li L, Wang Z, Yin MH, Li Q, Qi ZM (2017) Effect of early gait training on the functional rehabilitation after total knee arthroplasty. [Chinese]. Chinese Journal of Tissue Engineering Research 21:4288-4293 | Unable to Translate |
| Liao C-D, Lin L-F, Huang Y-C, Huang S-W, Chou L-C, Liou T-H (2015) Functional outcomes of outpatient balance training following total knee replacement in patients with knee osteoarthritis: a randomized controlled trial. Clin Rehabil 29:855-867 | Duplicate |
| Liao CD, Tsauo JY, Chiu YS, Ku JW, Huang SW, Liou TH (2019) Effects of elastic resistance exercise after total knee replacement on muscle mass and physical function in elderly women with osteoarthritis: a randomized controlled trial. Am J Phys Med Rehabil;10.1097/PHM.0000000000001344 | Duplicate |
| Liebs TR, Herzberg W, Rüther W, Russlies M, Hassenpflug J (2016) Quality-Adjusted Life Years Gained by Hip and Knee Replacement Surgery and Its Aftercare. Arch Phys Med Rehabil 97:691-700 | Duplicate |
| Liu M, Jia Y, Shi N (2018) Application value of an integrated treatment model of orthopedic rehabilitation in patients undergoing total knee arthroplasty. Int J Clin Exp Med 11:9455-9461 | Wrong Intervention |
| Liu PL, Li L, Zhang YK, Li M, Kane K, Wang YH, et al. (2009) A comparison of two rehabilitation protocols after simultaneous bilateral total knee arthroplasty: a controlled, randomized study. J Int Med Res 37:746-756 | Includes Passive Motion |
| Marcu IR, Patru S, Matei D, Bighea AC (2017) Role of physical exercise in patients with knee arthroplasty for osteoarthritis. Osteoporos Int 28:S330‐ | Abstract/Title Only |
| Moffet H, Tousignant M, Nadeau S, Merette C, Boissy P, Corriveau H, et al. (2011) Evaluating the quality of an on-going clinical trial on the effectiveness of telerehabilitation service after knee arthroplasty: a one-year summary. Physiotherapy (united kingdom). 97:eS821‐eS822 | Abstract/Title Only |
| Moutzouri M, Coutts F, Gliatis J, Billis E, Tsepis E, Gleeson N (2019) Early initiation of home-based sensori-motor training improves muscle strength, activation and size in patients after knee replacement: a secondary analysis of a controlled clinical trial. BMC Musculoskelet Disord 20:1-10 | Duplicate |
| Muto T, Kanemura N, Takayanagi K, Ogawa R, Tanikawa H, Okuma K (2015) Effects of multi-joint kinetics-chain exercise versus conventional exercise for patients with TKA: A randomized controlled trial. a 3-months research. Physiotherapy (United Kingdom) 101:eS1061 | Abstract/Title Only |
| Naylor JM, Crosbie J, Ko V (2015) IS THERE A ROLE FOR REHABILITATION STREAMING FOLLOWING TOTAL KNEE ARTHROPLASTY? PRELIMINARY INSIGHTS FROM A RANDOMIZED CONTROLLED TRIAL. Journal of Rehabilitation Medicine (Stiftelsen Rehabiliteringsinformation) 47:235-241 | Duplicate |
| [Nct (2008) Early Neuromuscular Electrical Stimulation For Quadriceps Muscle Activation Deficits Following Total Knee Replacement. https://clinicaltrials.gov/show/NCT00800254](https://clinicaltrials.gov/show/NCT00800254) | Duplicate |
| [Nct (2008) Effect of Physiotherapy After Total Knee Replacement. https://clinicaltrials.gov/show/NCT00807716](https://clinicaltrials.gov/show/NCT00807716) | Duplicate |
| [Nct (2009) Ergometer Cycling After Replacement of the Hip or Knee Joint. https://clinicaltrials.gov/show/NCT00951990](https://clinicaltrials.gov/show/NCT00951990) | Study not published yet |
| [Nct (2011) Early Progressive Strength Training to Patients With Unicompartmental Knee Replacement. https://clinicaltrials.gov/show/NCT01345825](https://clinicaltrials.gov/show/NCT01345825) | Study not published yet |
| [Nct (2011) The Difference Between Rehabilitation With or Without Strength Training After Total Knee Replacement. https://clinicaltrials.gov/show/NCT01351831](https://clinicaltrials.gov/show/NCT01351831) | Study Terminated |
| [Nct (2012) The HIHO Study: hospital Inpatient vs Home Rehabilitation After Total Knee Replacement. https://clinicaltrials.gov/show/NCT01583153](https://clinicaltrials.gov/show/NCT01583153) | Study not published yet |
| [Nct (2012) The Utilization of Nintendo Wii™ in Outpatient Rehabilitation Following Total Knee Replacement. https://clinicaltrials.gov/show/NCT01548664](https://clinicaltrials.gov/show/NCT01548664) | Study not published yet |
| [Nct (2013) Independent Exercise Compared With Formal Rehabilitation Following Primary Total Knee Replacement. https://clinicaltrials.gov/show/NCT01826305](https://clinicaltrials.gov/show/NCT01826305) | Wrong Study Design |
| [Nct (2014) Collaborative-care Intervention to Promote Physical Activity After Total Knee Arthroplasty. https://clinicaltrials.gov/show/NCT02075931](https://clinicaltrials.gov/show/NCT02075931) | Duplicate |
| [Nct (2014) Comparison of Treatments Following Total Knee Replacement. https://clinicaltrials.gov/show/NCT02237911](https://clinicaltrials.gov/show/NCT02237911) | Study not published yet |
| [Nct (2016) Virtual vs. Traditional Physical Therapy Following Total Knee Replacement. https://clinicaltrials.gov/show/NCT02914210](https://clinicaltrials.gov/show/NCT02914210) | Study not published yet |
| [Nct (2017) A Physical Therapist Administered Physical Activity Intervention After Total Knee Replacement. https://clinicaltrials.gov/show/NCT03228719](https://clinicaltrials.gov/show/NCT03228719) | Duplicate |
| [Nct (2017) Rehabilitation for Total Knee Replacement: a Novel Biofeedback System Versus Conventional Home-based Rehabilitation. https://clinicaltrials.gov/show/NCT03047252](https://clinicaltrials.gov/show/NCT03047252) | Duplicate |
| [Nct (2018) Effectiveness of a Community-based Tai Chi Rehabilitation Program for Patients After Total Knee Arthroplasty. https://clinicaltrials.gov/show/NCT03565380](https://clinicaltrials.gov/show/NCT03565380) | Duplicate |
| [Nct (2018) Virtual Reality Rehabilitation in Patients With Total Knee Replacement. https://clinicaltrials.gov/show/NCT03454256](https://clinicaltrials.gov/show/NCT03454256) | Duplicate |
| [Nct (2019) Effects of Exercise and Education in Patients With Chronic Pain After Total Knee Replacement. https://clinicaltrials.gov/show/NCT03886259](https://clinicaltrials.gov/show/NCT03886259) | Duplicate |
| [Nct (2020) Backwards Walking Programme Following Hip and Knee Arthroplasty. https://clinicaltrials.gov/show/NCT04247802](https://clinicaltrials.gov/show/NCT04247802) | Study not published yet |
| Negus JJ, Cawthorne DP, Chen JS, Scholes CJ, Parker DA, March LM (2015) Patient outcomes using Wii-enhanced rehabilitation after total knee replacement - the TKR-POWER study. Contemp Clin Trials 40:47‐53 | Wrong Study Design |
| Pesce V, Notarnicola A, Setti S, Moretti L, Vicenti G, Moretti B (2012) I-ONE therapy in patients undergoing total knee arthroplasty: Results at 12 months. J Orthop Traumatol 13 (SUPPL.1):S44 | Includes Passive Motion |
| Piva SR, Almeida G, Gil AB, Teixeira PE, Fitzgerald GK (2009) Effectiveness and feasibility of a balance training program post total knee arthroplasty: pilot randomized trial. J Orthop Sports Phys Ther 39:A29-30 | Abstract/Title Only |
| Piva SR, Catelani MB, Almeida GJ (2013) Comprehensive behavioral intervention compared to standard of care exercise program after total knee arthroplasty: A pilot randomized trial. Arthritis Rheum 65:S356 | Abstract/Title Only |
| Piva SR, Farrokhi S, Almeida G, Fitzgerald GK, Levison TJ, DiGioia AM (2015) Dose-Associated Changes in Gait Parameters in Response to Exercise Programs after Total Knee Arthroplasty: Secondary Analysis of Two Randomized Studies. International Journal of Physical Medicine & Rehabilitation 3:3-7 | Duplicate |
| Piva SR, Schneider M, Moore-Patterson C, Catelani MB, Gil A, Klatt B, et al. (2018) Randomized trial on exercise at late-stage after total knee replacement. Arthritis and Rheumatology 70:2185‐2186 | Duplicate |
| Potzelsberger B, Stoggl T, Lindinger SJ, Dirnberger J, Stadlmann M, Buchecker M, et al. (2015) Alpine Skiing With total knee ArthroPlasty (ASWAP): effects on strength and cardiorespiratory fitness. Scand J Med Sci Sports 25 Suppl 2:16-25 | Wrong Study Design |
| Pournajaf S, Goffredo M, Criscuolo S, Galli M, Damiani C, Franceschini M (2017) Virtual reality rehabilitation in patients with total knee replacement: Preliminary results. Gait Posture 57 (Supplement 3):17-18 | Abstract/Title Only |
| Room J, Batting M, Barker KL (2020) Development of a functional rehabilitation intervention for post knee arthroplasty patients: Community based Rehabilitation post Knee Arthroplasty (CORKA) trial. Physiotherapy 106:52-64 | Wrong Study Design |
| Room J, Newman M, Batting M, Kenealy N, Barker K (2019) Development of a functional rehabilitation intervention for post knee arthroplasty patients: COmmunity based Rehabilitation post Knee Arthroplasty (CORKA) trial. Physiotherapy (United Kingdom) 105 (Supplement 1):e160 | Abstract/Title Only |
| Rosal MC, Ayers D, Li W, Oatis C, Borg A, Zheng H, et al. (2011) A randomized clinical trial of a peri-operative behavioral intervention to improve physical activity adherence and functional outcomes following total knee replacement. BMC Musculoskelet Disord 12:226-226 | Wrong Intervention |
| Russell TG, Buttrum P, Wootton R, Jull GA (2003) Low-bandwidth telerehabilitation for patients who have undergone total knee replacement: preliminary results. J Telemed Telecare 9:S2:44-47 | Duplicate |
| Russo LR, Benedetti MG, Mariani E, Roberti di Sarsina T, Zaffagnini S (2017) The Videoinsight Method: improving early results following total knee arthroplasty. Knee Surg Sports Traumatol Arthrosc 25:2967‐2971 | Duplicate |
| Sindhupakorn B, Numpaisal P-o, Thienpratharn S, Jomkoh D (2019) A home visit program versus a non-home visit program in total knee replacement patients: a randomized controlled trial. J Orthop Surg Res 14: | Wrong Intervention |
| Steurer J (2017) Praxis 106:841-842 | Wrong Study Design |
| Stevens JE (2002) Physiologically-based rehabilitation for patients after total knee arthroplasty. 157 p-157 p | Wrong Study Design |
| Stocker B, Babendererde C, Rohner-Spengler M, Muller UW, Meichtry A, Luomajoki H (2018) Pflege 31:19-29 | Wrong Intervention |
| Szöts K, Konradsen H, Solgaard S, Østergaard B (2016) Telephone Follow-Up by Nurse After Total Knee Arthroplasty: results of a Randomized Clinical Trial. Orthopedic nursing 35:411‐420 | Wrong Intervention |
| Tennent DJ, Hylden CM, Johnson AE, Burns TC, Wilken JM, Owens JG (2017) Blood flow restriction training after knee arthroscopy: A randomized controlled pilot study. Clin J Sport Med 27:245-252 | Wrong Patient Population |
| Thiengwittayaporn S, Kangkano N, Krishnamra N, Charoenphandhu N (2017) Comparison between diary-actuated rehabilitation program and conventional physical therapy on mobility and function following total knee arthroplasty. Osteoporos Int 28 (Supplement 1):S194-S195 | Abstract/Title Only |
| Valtonen A, Pöyhönen T, Sipilä S, Heinonen A (2011) Maintenance of aquatic training-induced benefits on mobility and lower-extremity muscles among persons with unilateral knee replacement. Arch Phys Med Rehabil 92:1944‐1950 | Duplicate |
| van Eeden FM, van Halewijn KF, Swart NM, Festen DA, Bily W, Franz C, et al. (2016) Efficacy and Safety of Leg-Press Training With Moderate Vibration After Total Knee Arthroplasty Remains Unclear...Bily W, Franz C, Trimmel L, Loefler S, Cvecka J, Zampieri S, Kasche W, Sarabon N, Zenz P, Kern H. Effects of Leg-Press Training With Moderate Vibration on Muscle Strength, Pain, and Function After Total Knee Arthroplasty: A Randomized Controlled Trial. Arch Phys Med Rehabil. 2016 Jun;97(6):857-65. Arch Phys Med Rehabil 97:2018-2019 | Wrong Study Design |
| Villafañe JH, Isgrò M, Borsatti M, Berjano P, Pirali C, Negrini S (2017) Effects of action observation treatment in recovery after total knee replacement: a prospective clinical trial. Clin Rehabil 31:361-368 | Includes Passive Motion |
| Weaver FM, Hughes SL, Almagor O, Wixson R, Manheim L, Fulton B, et al. (2003) Comparison of two home care protocols for total joint replacement. J Am Geriatr Soc 51:523-528 | Wrong Intervention |
| Weber-Spickschen S, Hardt S, Horstmann H, Krettek C (2018) Improved early recovery after TKA through an app-based, feedback-controlled active muscle training-A prospective randomized trial. Orthopaedic Journal of Sports Medicine. Conference: 92nd DKG Annual Meeting. Germany. 6: | Abstract/Title Only |
| Wylde V, Artz N, Dixon S, Marques E, Lenguerrand E, Blom A, et al. (2014) Effectiveness and cost-effectiveness of a group-based outpatient physiotherapy intervention following knee replacement for osteoarthritis: feasibility study for a randomised controlled trial. Osteoarthritis Cartilage 22:S433‐ | Abstract/Title Only |
