# Supplementary material for: Reporting of post-operative rehabilitation interventions for Total knee arthroplasty: a scoping review
Source: BMC Musculoskelet Disord. 2021 Jun 30;22:602. doi: 10.1186/s12891-021-04460-w (PMC8247251; doi:10.1186/s12891-021-04460-w)
Supplement: Supplementary file 5 — Additional file 5: Appendix E. CERT Analysis. [file 12891_2021_4460_MOESM5_ESM.docx]

## Appendix E – CERT Analysis

| **Author** | **Intervention** | **Equipment** | **Qualifications** | **Individual or Group** | **Supervised or Unsupervised** | **Adherence Measurements** | **Motivation** | **Decision for Progression** | **Description of Progression** | **Exercise Description** | **Home Component** | **Non-exercise** | **Adverse Event** | **Setting** | **Dosage** | **Generic or Tailored** | **Description of Tailoring** | **Start Decision** | **Planned Delivery** | **Adherence/Fidelity** |
| --- | --- | --- | --- | --- | --- | --- | --- | --- | --- | --- | --- | --- | --- | --- | --- | --- | --- | --- | --- | --- |
| Akbaba et al. (2016) | Less Supervision | + | ? | + | + | ? | + | + | + | + | + | + | + | + | ? | + | ? | ? | ? | ? |
|  | Healthy Control | N/A | N/A | N/A | N/A | N/A | N/A | N/A | N/A | N/A | N/A | N/A | N/A | N/A | N/A | N/A | N/A | N/A | N/A | N/A |
|  | Physical Therapy | + | ? | + | + | ? | + | + | + | + | + | + | + | + | + | + | ? | ? | ? | ? |
| Alghadir et al. (2016) | Pre-and-Post Exercise | ? | ? | + | + | ? | ? | ? | ? | ? | + | + | ? | + | ? | + | N/A | ? | ? | ? |
|  | Post-Exercise Only | ? | ? | + | + | ? | ? | ? | ? | ? | + | + | ? | + | ? | + | N/A | ? | ? | ? |
| Anneli et al. (2017) | Exercise Group | + | ? | + | + | ? | + | + | + | + | + | + | + | + | + | + | N/A | ? | ? | + |
|  | Control | ? | ? | + | + | ? | + | + |  | ? | + | + | ? | + | ? | + | N/A | ? | ? | + |
| Aprile et al. (2011) | Individual followed by Group Exercise | ? | ? | + | + | ? | ? | ? | ? | ? | ? | ? | ? | + | ? | + | N/A | + | ? | ? |
|  | Group followed by Individual Exercise | ? | ? | + | + | ? | ? | ? | ? | ? | ? | ? | ? | + | ? | + | N/A | + | ? | ? |
| Artz et al. (2017) | Physiotherapy intervention | ? | ? | + | + | ? | ? | ? | ? | ? | + | + | ? | + | ? | + | N/A | ? | ? | ? |
|  | Usual Care | ? | ? | + | + | ? | ? | ? | ? | ? | + | + | ? | + | ? | + | N/A | ? | ? | ? |
| Avramidis et al. (2011) | Electric Muscle Stimulation | ? | + | + | + | ? | ? | ? | ? | + | ? | + | ? | + | + | + | N/A | + | ? | + |
|  | Usual Care | ? | ? | + | + | ? | ? | ? | ? | + | ? | ? | ? | + | ? | + | N/A | + | ? | + |
| Bade et al. (2017) | High-Intensity Exercise | ? | ? | + | + | + | + | + | + | + | + | + | + | + | + | + | N/A | + | + | + |
|  | Low-Intensity Exeercise | + | + | + | + | + | ? | + | + | + | + | + | + | + | + | + | N/A | + | + | + |
| Bellelli et al. (2010) | Standard + Video with motor exercises | ? | ? | + | + | ? | ? | + | + | + | ? | ? | ? | + | ? | + | N/A | + | ? | ? |
|  | Standard + Video without motor | ? | ? | + | + | ? | ? | + | + | + | ? | + | ? | + | ? | + | N/A | + | ? | ? |
| Bily et al. (2016) | Leg-Press | + | ? | + | + | + | ? | + | ? | + | ? | ? | + | + | + | + | N/A | ? | + | ? |
|  | Functional Physiotherapy | + | ? | + | + | + | ? | + | ? | + | ? | + | + | + | + | + | ? | + | + | ? |
| Bini et al. (2017) | Asynchronous Video | ? | ? | + | + | + | ? | + | + | ? | + | + | ? | + | + | + | ? | + | ? | + |
|  | Standard Care | ? | ? | + | + | ? | ? | ? | ? | ? | + | ? | ? | + | ? | ? | ? | + | ? | + |
| Bohl et al. (2019) | PT 0-Day Post-op | ? | ? | + | + | ? | ? | + | ? | ? | ? | ? | ? | + | ? | + | + | + | ? | ? |
|  | PT 1-Day Post Op |  | ? | + | + | ? | ? | ? | ? | ? | ? | ? | ? | + | ? | + | + | + | ? | ? |
| Brandes et al. (2018) | Standard Care + Activity Counselling | ? | + | + | + | + | + | + | ? | ? | ? | + | ? | ? | + | + | + | + | + | + |
|  | Standard Care | ? | ?? | + | + | + | ? | ? | ? | ? | ? | + | ? | + | + | + | + | + | + | + |
| Bruun-Olsen et al. (2013) | Walking Skill | + | ? | + | + | ? | + | + | ? | + | ? | ? | ? | + | ? | + | ? | + | ? | ? |
|  | Standard Care | ? | ? | + | + | ? | ? | ? | ? | ? | ? | ? | ? | + | ? | + | ? | + | ? | ? |
| Bugbee et al. (2016) | AlterG | + | + | + | + | ? | ? | + | ? | + | + | + | + | + | + | + | + | + | ? | + |
|  | Land-Based | + | + | + | + | ? | ? | + | ? | ? | + | + | + | + | + | + | + | + | ? | + |
| Buhaglar et al. (2017) | Inpatient with Home Program | + | ? | + | + | + | + | + | ? | + | + | ? | + | + | ? | + | N/A | + | + | ? |
|  | Home Program | + | ? | + | + | + | + | + | ? | + | + | ? | + | + | + | + | N/A | + | + | ? |
|  | Observational Group | + | ? | + | + | + | + | + | ? | + | + | ? | + | + | + | + | N/A | + | + | ? |
| Bulthuis et al. (2007) | Intensive Exercise | ? | ? | + | + | ? | ? | + | + | ? | ? | + | ? | + | ? | + | + | + | ? | ? |
|  | Standard Care | ? | ? | ? | ? | ? | ? | ? | ? | ? | ? | + | ? | + | ? | ? | ? | ? | ? | ? |
| Cai et al. (2017) | Cognitive Behavioural Intervention | ? | ? | + | + | ? | ? | + | + | + | ? | + | + | + | + | + | ? | + | ? | + |
|  | Control | ? | + | + | + | ? | ? | + | + | + | ? | + | + | + | + | + | ? | + | ? | + |
| Cai et al. (2018) | Cognitive Behavioural Intervention | ? | ? | + | + | ? | ? | + | + | ? | ? | + | ? | + | ? | + | ? | + | ? | ? |
|  | Standard Care | ? | ? | + | + | ? | ? | ? | ? | ? | ? | + | ? | ? | ? | + | N/A | ? | ? | ? |
| Campbell et al. (2019) | SMS bot | ? | ? | + | + | + | + | ? | ? | ? | + | + | + | + | ? | + | N/A | ? | + | ? |
|  | Traditional Education | ? | ? | + | + | + | + | ? | ? | ? | + | + | + | + | ? | + | N/A | ? | + | ? |
| Chen et al. (2017) | EMG Biofeedback | + | ? | + | + | ? | ? | + | + | + | ? | ? | ? | + | ? | + | ? | + | ? | ? |
|  | Standard Care | ? | ? | + | + | ? | ? | + | + | + | ? | ? | ? | + | + | + | N/A | ? | ? | ? |
|  | Joint Rehabilitation | + | ? | + | + | ? | ? | + | + | + | ? | ? | ? | + | ? | + | N/A | + | ? | ? |
| Chen et al. (2016) | Telephone Call + Exercise | ? | + | + | + | + | + | + | ? | ? | + | ? | ? | + | ? | + | N/A | + | + | + |
|  | Exercise Only | ? | + | + | + | + | + | + | ? | ? | + | ? | ? | + | ? | + | N/A | + | + | + |
| Christiansen et al. (2015) | Weight-Bearing Biofeedback | + | ? | + | + | + | + | + | + | ? | + | + | ? | + | ? | + | ? | + | + | ? |
|  | Standard Care | + | ? | + | + | + | + | + | + | ? | + | + | ? | + | ? | + | ? | + | + | ? |
| Christiansen et al. (2020) | Fitbit/Step Goals | + | ? | + | + | + | + | + | + | + | + | + | + | + | ? | + | N/A | ? | + | + |
|  | Standard Care | + | ? | + | + | + | + | + | + | + | + | + | + | + | ? | + | N/A | ? | + | + |
| De Fine et al. (2017) | High-flexion group | ? | ? | + | + | ? | ? | ? | ? | ? | ? | + | + | + | ? | ? | ? | ? | ? | ? |
|  | Mid-flexion group | ? | ? | + | ? | ? | ? | ? | ? | ? | ? | + | + | + | ? | ? | ? | ? | ? | ? |
| Debbi et al. (2019) | Biomechanical device | + | ? | + | + | ? | ? | + | + | ? | + | ? | + | + | ? | ? | ? | + | + | ? |
|  | Sham Device | + | ? | + | + | ? | ? | + | + | ? | + | ? | + | + | ? | ? | ? | + | + | ? |
| Demircioglu et al. (2015) | NMES | ? | ? | + | + | ? | ? | ? | ? | ? | + | + | + | + | ? | + | N/A | + | ? | ? |
|  | Exercise Group | ? | ? | + | + | ? | ? | ? | ? | ? | + | + | + | + | ? | + | N/A | + | ? | ? |
| den Hertog et al. (2012) | Fast-Track | ? | ? | + | + | ? | + | ? | ? | ? | ? | ? | + | + | ? | + | N/A | + | ? | ? |
|  | Standard Care | ? | ? | + | + | ? | ? | + | + | ? | ? | + | + | + | ? | + | ? | + | ? | ? |
| Doerfler et al. (2016) | High-Velocity Exercise | + | ? | + | + | ? | + | + | + | + | + | + | ? | + | ? | + | N/A | + | ? | ? |
|  | Slow-Velocity Exercise | + | ? | + | + | ? | + | + | + | + | + | + | ? | + | + | + | N/A | + | ? | ? |
| Donec & Krisciunas (2014) | Standard Care + KTape | ? | ? | + | + | ? | ? | ? | ? | ? | ? | + | ? | + | ? | + | N/A | ? | ? | ? |
|  | Standard Care | ? | ? | + | + | ? | ? | ? | ? | ? | ? | + | ? | + | ? | + | N/A | ? | ? | ? |
| Ebert et al. (2013) | Manual Lymphatic Drainage | ? | ? | + | + | ? | ? | ? | ? | + | ? | + | ? | + | ? | + | N/A | + | ? | ? |
|  | Control | ? | ? | + | + | ? | ? | ? | ? | + | ? | + | ? | + | ? | + | N/A | + | ? | ? |
| Eichler et al. (2019) | Telerehab | ? | ? | + | + | + | + | ? | ? | ? | + | ? | ? | + | ? | + | ? | ? | + | + |
|  | Standard Care | ? | ? | ? | + | ? | ? | ? | ? | ? | ? | ? | ? | + | ? | ? | ? | ? | ? | + |
| Eisermann et al. (2004) | Computer-aided Training | + | ? | + | + | ? | + | ? | ? | ? | ? | + | + | + | ? | + | + | ? | ? | ? |
|  | Standard Care | + | ? | + | + | ? | ? | ? | ? | ? | ? | + | + | + | ? | + | N/A | ? | ? | ? |
| Evgeniadis et al. (2008) | Pre-op Only | + | ? | + | + | ? | ? | + | + | ? | + | ? | ? | + | ? | + | ? | + | ? | ? |
|  | No Treatment | N/A | N/A | N/A | N/A | N/A | N/A | N/A | N/A | N/A | N/A | N/A | N/A | N/A | N/A | N/A | N/A | N/A | N/A | N/A |
|  | Post-op only | + | ? | + | + | ? | ? | + | ? | ? | + | ? | ? | + | ? | + | ? | + | ? | ? |
| Ficklscherer et al. (2016) | Standard Care + Nintendo Wii | + | ? | + | + | ? | + | ? | ? | ? | ? | + | ? | + | ? | ? | ? | ? | ? | ? |
|  | Standard Care | ? | ? | ? | ? | ? | ? | ? | ? | ? | ? | + | ? | ? | ? | ? | ? | ? | ? | ? |
| Fleischman et al. (2019) | Web-based PT | ? | ? | + | + | ? | ? | + | ? | ? | + | ? | + | + | ? | + | N/A | + | ? | ? |
|  | Outpatient PT | ? | ? | ? | + | ? | ? | ? | ? | ? | + | ? | + | + | ? | ? | ? | ? | ? | ? |
|  | Home Exercise | ? | ? | + | + | ? | ? | + | ? | ? | + | ? | + | + | ? | + | N/A | + | ? | ? |
| Fortuno Godes et al. (2010) | PT + Physiologic Life Rhythm | ? | ? | ? | ? | ? | ? | ? | ? | + | ? | + | ? | ? | ? | ? | ? | ? | ? | ? |
|  | Physical Therapy | ? | ? | ? | ? | ? | ? | ? | ? | + | ? | ? | + | ? | ? | ? | ? | ? | ? | ? |
| Fransen et al. (2017) | Group Exercise | + | ? | + | + | + | ? | + | + | + | + | ? | + | + | + | + | N/A | + | + | + |
|  | Standard Care | + | ? | ? | ? | ? | ? | ? | ? | ? | ? | ? | + | + | ? | ? | ? | ? | ? | + |
| Frost et al. (2002) | Functional Training | + | ? | + | + | ? | ? | + | + | + | + | + | ? | + | ? | + | N/A | ? | ? | ? |
|  | Standard Care | + | ? | + | + | ? | ? | ? | ? | + | + | ? | ? | + | ? | + | N/A | ? | ? | ? |
| Fung et al. (2012) | Wii Fit Gaming | + | ? | + | + | ? | ? | + | ? | + | ? | ? | ? | + | ? | + | N/A | + | ? | ? |
|  | Lower Extremity Exercise | N/A | ? | ? | + | ? | ? | + | + | + | ? | ? | ? | + | ? | + | N/A | ? | ? | ? |
| Gianola et al. (2020) | Virtual Reality | + | ? | + | + | ? | ? | ? | ? | + | ? | ? | ? | + | + | + | N/A | ? | ? | ? |
|  | Standard Care | + | ? | + | + | ? | ? | ? | ? | + | ? | ? | ? | + | ? | + | N/A | ? | ? | ? |
| Giaquinto et al. (2010) | Hydrotherapy | ? | ? | ? | + | ? | ? | ? | ? | ? | ? | + | + | + | ? | + | N/A | ? | ? | ? |
|  | Land-Based | + | ? | ? | + | ? | ? | ? | ? | ? | ? | + | + | + | ? | + | N/A | ? | ? | ? |
| Han et al. (2015) | Home Exercise | + | ? | + | + | ? | + | + | + | ? | + | + | + | + | ? | + | N/A | + | ? | ? |
|  | Standard Care | ? | ? | + | + | ? | ? | ? | ? | ? | ? | ? | + | + | ? | ? | ? | + | ? | ? |
| Hardt et al. (2018) | Standard Care + App-Based Feedback | ? | ? | + | + | ? | ? | ? | ? | + | ? | + | ? | + | ? | + | ? | + | ? | ? |
|  | Standard Care | ? | ? | + | + | ? | ? | ? | ? | ? | ? | + | ? | + | ? | + | N/A | ? | ? | ? |
| Harikesavan et al. (2017) | Standard Care + Hip Strengthening | + | ? | + | + | ? | + | + | + | + | + | + | ? | + | + | + | N/A | ? | ? | ? |
|  | Standard Care | + | ? | + | + | ? | + | + | + | + | + | + | ? | + | + | + | N/A | ? | ? | ? |
| Harmer et al. (2009) | Water-Based | ? | ? | + | + | + | + | ? | ? | ? | + | ? | + | + | ? | + | N/A | ? | + | ? |
|  | Land-Based | + | ? | + | + | + | + | ? | ? | ? | + | ? | + | + | ? | + | N/A | ? | + | ? |
| Hepperger et al. (2017) | Guided Hiking | + | ? | + | + | + | ? | + | + | + | ? | ? | ? | + | ? | + | N/A | ? | + | ? |
|  | Continue with ADL's | ? | N/A | + | + | ? | ? | N/A | N/A | ? | + | ? | ? | + | ? | ? | ? | N/A | ? | ? |
| Hoorntje et al. (2020) | Goal Attainment Scaling | ? | ? | + | + | + | ? | ? | ? | + | ? | ? | ? | ? | ? | + | ? | ? | + | ? |
|  | Standard Care | ? | ? | ? | + | 0 | ? | ? | ? | ? | ? | ? | ? | + | ? | + | N/A | ? | + | ? |
| Husby et al. (2018) | Maximal Strength Training | + | ? | + | + | ? | ? | + | + | + | ? | + | ? | + | + | + | N/A | ? | ? | ? |
|  | Standard Care | ? | ? | + | + | ? | + | ? | ? | ? | + | + | ? | + | ? | ? | N/A | ? | ? | ? |
| Iwakiri et al. (2020) | ROM Day 1 | ? | ? | + | + | ? | ? | ? | ? | ? | ? | ? | + | + | ? | + | N/A | ? | ? | ? |
|  | ROM Day 7 | ? | ? | + | + | ? | ? | ? | ? | ? | ? | ? | + | + | ? | + | N/A | ? | ? | ? |
| Jakobsen et al. (2014) | Physical Strength Training | + | + | + | + | ? | ? | + | + | + | + | + | + | + | + | + | N/A | ? | ? | ? |
|  | Standard Care | + | + | + | + | ? | ? | + | + | + | + | + | + | + | + | + | N/A | ? | ? | ? |
| Jin et al. (2018) | Virtual Reality | + | ? | + | + | ? | + | ? | ? | ? | ? | + | ? | + | ? | + | N/A | ? | ? | ? |
|  | Standard Care | ? | ? | + | + | ? | ? | ? | ? | ? | ? | + | ? | + | + | + | N/A | ? | ? | ? |
| Jogi et al. (2015) | Standard Care + Balance | + | ? | + | + | ? | ? | + | ? | + | + | + | ? | + | + | + | N/A | + | + | ? |
|  | Standard Care | + | ? | + | + | ? | ? | + | ? | + | + | + | ? | + | ? | + | N/A | + | + | ? |
| Johnson et al. (2010) | Whole Body Vibration | + | ? | + | + | + | ? | + | + | + | + | + | + | + | + | + | N/A | + | + | ? |
|  | Progressive Resistance Training | + | ? | + | + | + | ? | + | + | + | + | + | + | + | + | + | N/A | + | + | ? |
| Ju et al. (2019) | Strength Training + Moxibustion Therapy | ? | ? | ? | + | ? | ? | ? | ? | + | ? | + | + | ? | + | + | N/A | ? | ? | ? |
|  | Strength Training | ? | ? | ? | + | ? | ? | ? | ? | + | ? | ? | + | ? | + | + | N/A | ? | ? | ? |
| Karaman et al. (2017) | Pilates | + | ? | + | + | ? | + | ? | ? | + | + | + | ? | + | ? | + | N/A | ? | ? | ? |
|  | Standard Care | ? | ? | + | + | ? | + | + | + | + | + | + | ? | + | + | + | N/A | ? | ? | ? |
| Kauppila et al. (2010) | Multidisciplinary Rehab | + | ? | + | + | ? | + | + | + | + | + | + | + | + | ? | + | N/A | + | ? | + |
|  | Standard Care | ? | ? | ? | + | ? | ? | ? | ? | + | + | + | ? | + | ? | + | ? | ? | ? | + |
| Kelly et al. (2016) | High Velocity | + | ? | + | + | + | ? | + | + | + | + | + | + | + | + | + | N/A | + | + | ? |
|  | Low Velocity | + | ? | + | + | + | ? | + | + | + | + | + | + | + | + | + | N/A | + | + | ? |
| Kramer et al. (2003) | Clinic-Based | ? | ? | + | + | + | ? | ? | + | ? | + | + | ? | + | + | + | N/A | + | + | ? |
|  | Home Based | ? | ? | + | + | + | + | ? | ? | ? | + | + | ? | + | ? | + | N/A | + | + | ? |
| Lee et al. (2020) | Dynamic Exercise Visual Feedback | ? | ? | + | + | ? | ? | + | + | + | ? | ? | ? | + | + | + | N/A | ? | ? | ? |
|  | Dynamic Exercise | ? | ? | + | + | ? | ? | + | + | + | ? | ? | ? | + | + | + | N/A | ? | ? | ? |
| Lenguerrand et al. (2019) | Physical Therapy | + | ? | + | + | + | + | + | + | + | + | ? | + | + | + | + | + | ? | + | + |
|  | Standard Care | ? | ? | + | + | ? | ? | ? | ? | ? | ? | ? | + | + | ? | ? | ? | ? | ? | + |
| Lenssen et al. (2006) | 2 PT Sessions/day | ? | ? | + | + | + | ? | ? | ? | + | ? | ? | ? | + | ? | + | N/A | ? | ? | ? |
|  | 1 PT Session/day | ? | ? | + | + | + | ? | ? | ? | + | ? | ? | ? | + | ? | + | N/A | ? | ? | ? |
| Levine et al. (2013) | Physical Therapy | ? | ? | + | + | ? | ? | ? | ? | ? | ? | ? | ? | ? | ? | + | N/A | ? | ? | ? |
|  | NMES | ? | ? | + | + | ? | ? | ? | ? | ? | ? | + | ? | ? | ? | + | N/A | ? | ? | ? |
| Li et al. (2019) | Tai Chi Chuan | ? | + | + | + | ? | ? | ? | ? | + | ? | ? | + | ? | ? | + | N/A | + | ? | + |
|  | Standard Care | ? | ? | + | + | ? | ? | ? | ? | + | ? | ? | + | ? | ? | + | N/A | ? | ? | + |
| Liao et al. (2013) | Functional Training + Balance | + | ? | + | + | ? | ? | ? | ? | + | ? | ? | ? | + | + | + | N/A | ? | ? | ? |
|  | Functional Training | ? | ? | + | + | ? | ? | ? | ? | + | ? | ? | ? | + | + | + | N/A | ? | ? | ? |
| Liao et al. (2020) | Elastic Resistance Exercise | + | ? | + | + | + | ? | + | + | + | + | ? | + | + | + | + | N/A | ? | + | + |
|  | Standard Care | ? | ? | + | + | + | ? | ? | ? | ? | ? | ? | + | + | ? | + | N/A | ? | + | + |
| Liebs et al. (2010) | Ergometric Cycling | + | ? | + | + | ? | ? | ? | ? | + | ? | + | + | ? | ? | + | N/A | ? | ? | + |
|  | Standard Care | ? | ? | + | + | ? | ? | ? | ? | ? | ? | + | + | ? | ? | + | N/A | ? | ? | + |
| Liebs et al. (2012) | Early Aquatics | + | ? | ? | + | ? | ? | ? | ? | ? | ? | + | + | + | ? | ? | ? | ? | ? | ? |
|  | 14 Day Post-Op Aquatics | + | ? | ? | + | ? | ? | ? | ? | ? | ? | + | + | + | ? | ? | ? | ? | ? | ? |
| Lin et al. (2018) | Lower Extremity Muscle Strength Training | + | + | + | + | ? | + | ? | ? | + | ? | + | + | + | + | + | N/A | ? | ? | + |
|  | Standard Care | N/A | ? | + | + | ? | + | ? | ? | ? | + | ? | + | + | ? | + | N/A | ? | ? | + |
| Lowe et al. (2012) | Home Visit | + | ? | + | + | + | ? | + | + | + | + | + | + | + | ? | + | N/A | + | + | ? |
|  | Physiotherapy |  | ? | + | + | ? | + | + | + | + | + | + | + | + | ? | + | N/A | + | ? | ? |
| Lysack et al. (2005) | Standard Care + 1 App Based Exercise | ? | ? | + | + | + | ? | ? | ? | ? | ? | ? | ? | + | ? | + | N/A | ? | + | ? |
|  | Standard Care | ? | ? | + | + | + | ? | ? | ? | ? | ? | ? | ? | + | ? | + | N/A | ? | + | ? |
| Madsen et al. (2013) | Group Based Exercise | + | ? | + | + | + | + | + | + | + | + | ? | ? | + | + | + | N/A | ? | + | ? |
|  | Home Based Exercise | ? | ? | + | + | + | ? | ? | ? | ? | + | ? | ? | + | ? | + | N/A | ? | + | ? |
| Mahomed et al. (2008) | Inpatient Rehab | ? | ? | + | + | + | ? | + | ? | + | ? | + | ? | + | ? | + | N/A | ? | + | ? |
|  | Home Based Exercise | ? | ? | + | + | + | ? | + | + | + | ? | + | + | + | ? | + | N/A | + | + | ? |
| McAvoy (2009) | Land Physical therapy | + | ? | + | + | ? | ? | ? | ? | + | ? | + | ? | + | + | + | N/A | ? | ? | ? |
|  | Integrated Physical Therapy | ? | ? | + | + | ? | ? | ? | ? | + | ? | + | ? | + | + | + | N/A | ? | ? | ? |
| Mitchell et al. (2005) | Usual Hospital Physiotherapy | ? | ? | + | + | ? | ? | ? | ? | ? | ? | + | ? | + | ? | + | N/A | ? | ? | ? |
|  | Home Based Exercise | ? | ? | + | + | ? | ? | ? | ? | ? | ? | + | ? | + | ? | + | N/A | ? | ? | ? |
| Mockford et al. (2008) | Outpatient Physiotherapy | ? | ? | + | + | + | ? | ? | ? | + | + | ? | ? | ? | ? | + | N/A | ? | ? | ? |
|  | Standard Care | ? | ? | + | + | ? | ? | ? | ? | + | + | ? | ? | ? | ? | + | N/A | ? | ? | ? |
| Moffet et al. (2004) | Intensive Functional Rehab | + | ? | + | + | + | ? | + | ? | + | + | + | + | + | + | + | + | + | + | + |
|  | Standard Care | ? | ? | + | + | ? | ? | ? | ? | + | + | + | + | + | ? | + | N/A | ? | ? | + |
| Moffet et al. (2015) | Telerehab | ? | ? | + | + | + | ? | + | ? | ? | + | + | + | + | ? | + | ? | ? | + | ? |
|  | Face-to-Face | ? | ? | + | + | + | ? | + | ? | ? | + | + | + | + | ? | + | ? | ? | + | ? |
| Monticone et al. (2013) | Functional Training | + | ? | + | + | ? | + | ? | ? | + | + | + | + | + | ? | + | N/A | ? | ? | ? |
|  | Standard Care | + | ? | + | + | ? | + | ? | ? | + | + | + | + | + | ? | + | N/A | ? | ? | ? |
| Moutzouri et al. (2018) | Focal Sensorimotor Exercise Training | + | ? | + | + | ? | ? | + | + | + | + | ? | ? | + | + | + | ? | ? | ? | ? |
|  | Functional Training | + | ? | + | + | ? | ? | + | + | + | + | ? | + | + | + | + | ? | ? | ? | ? |
| Munin et al. (1998) | 3-days post-op | ? | ? | + | + | ? | ? | ? | ? | ? | ? | + | ? | + | ? | + | N/A | ? | ? | ? |
|  | 7-days post-op | ? | ? | + | + | ? | ? | ? | ? | ? | ? | + | ? | + | ? | + | N/A | ? | ? | ? |
| Ko et al. (2013) | One-on-One Exercises | ? | ? | + | + | + | ? | + | + | ? | + | + | + | + | ? | ? | ? | ? | + | + |
|  | Group Based | + | ? | + | + | + | ? | + | + | + | + | ? | + | + | + | + | N/A | ? | + | + |
|  | Monitored Home Exercise | + | ? | + | + | + | ? | + | + | + | + | + | + | + | + | ? | ? | ? | + | + |
| Paravlic et al. (2019) | Motor Imagery | + | ? | + | + | + | + | ? | ? | + | + | + | + | + | ? | + | + | ? | + | ? |
|  | Standard Care | N/A | ? | + | + | + | + | ? | ? | + | + | ? | + | + | ? | + | + | ? | + | ? |
| Paxton et al. (2018) | Physical Activity Feedback | ? | ? | + | + | + | + | + | + | ? | ? | + | ? | + | ? | + | N/A | + | + | ? |
|  | Standard Care | ? | ? | + | + | ? | + | ? | ? | ? | ? | ? | ? | + | ? | + | N/A | + | ? | ? |
| Peiris et al. (2012) | OT + PT 6 days | ? | ? | + | + | + | ? | ? | ? | ? | ? | ? | ? | + | ? | ? | ? | ? | ? | ? |
|  | OT + PT 5 days | ? | ? | + | + | + | ? | ? | ? | ? | ? | ? | ? | + | ? | ? | ? | ? | ? | ? |
| Petterson et al. (2009) | Exercise + NMES | + | ? | + | + | ? | ? | ? | ? | + | ? | + | + | + | + | + | N/A | ? | ? | ? |
|  | Exercise Only | + | ? | + | + | ? | ? | ? | ? | + | ? | + | + | + | + | + | N/A | ? | ? | ? |
| Piqueras et al. (2013) | Telerehab | ? | ? | + | + | ? | ? | ? | ? | ? | + | ? | ? | + | ? | ? | ? | ? | ? | ? |
|  | Standard Care | ? | ? | + | + | ? | ? | ? | ? | ? | + | ? | + | + | ? | ? | ? | ? | ? | ? |
| Piva et al. (2019) | Physical Therapy | ? | ? | + | + | ? | ? | + | + | + | + | ? | ? | + | ? | + | ? | + | ? | ? |
|  | Community Care | ? | ? | + | + | ? | ? | ? | ? | ? | ? | ? | + | + | ? | + | N/A | + | ? | ? |
|  | No Treatment | N/A | N/A | N/A | N/A | N/A | N/A | N/A | N/A | N/A | N/A | N/A | N/A | N/A | N/A | N/A | N/A | N/A | N/A | N/A |
| Piva et al. (2017) | Cognitive Behavioural Intervention | + | ? | + | + | + | + | + | + | + | + | + | ? | + | + | + | N/A | + | + | ? |
|  | Standard Care | + | ? | + | + | + | ? | + | + | + | + | ? | + | + | + | + | N/A | ? | + | ? |
| Piva et al. (2010) | Functional Training + Balance | + | ? | + | + | + | + | + | + | + | + | ? | ? | + | + | + | N/A | ? | + | ? |
|  | Functional Training | + | ? | + | + | + | + | + | + | + | + | ? | + | + | + | + | N/A | ? | + | ? |
| Prvu Bettger et al. (2020) | Telerehab | + | ? | + | + | + | + | + | ? | ? | + | ? | + | + | ? | + | ? | ? | + | ? |
|  | Standard Care | ? | ? | ? | ? | + | ? | ? | ? | ? | ? | ? | ? | ? | ? | + | N/A | ? | + | ? |
| Rahmann et al. (2009) | Ward Exercise | + | + | + | + | ? | ? | + | + | + | ? | ? | + | + | + | + | N/A | + | ? | + |
|  | Aquatic Physio | + | + | + | + | ? | ? | + | + | + | ? | ? | + | + | + | + | N/A | + | ? | + |
|  | Water Exercises | + | + | + | + | ? | ? | + | + | + | ? | ? | + | + | + | + | N/A | + | ? | + |
| Rajan et al. (2004) | Outpatient/Inpatient PT | ? | ? | ? | + | ? | ? | ? | ? | ? | + | ? | ? | + | ? | ? | ? | + | ? | ? |
|  | Inpatient Only | ? | ? | + | + | ? | ? | ? | ? | ? | + | ? | ? | + | ? | ? | ? | + | ? | ? |
| Roig-Casasus et al. (2018) | Balance Training + Functional | + | ? | + | + | ? | ? | ? | ? | + | ? | ? | + | + | + | + | N/A | + | ? | ? |
|  | Functional Training | ? | ? | + | + | ? | ? | ? | ? | + | ? | ? | + | + | + | + | N/A | + | ? | ? |
| Russell et al. (2011) | Telerehab | + | ? | + | + | + | + | ? | ? | ? | + | ? | ? | + | ? | + | N/A | ? | + | ? |
|  | Face-to-Face | ? | ? | + | + | + | + | ? | ? | ? | + | ? | ? | + | ? | + | N/A | ? | + | ? |
| Russo et al. (2017) | Videoinsight group | ? | ? | + | + | ? | + | ? | ? | ? | + | ? | ? | ? | ? | ? | ? | ? | ? | ? |
|  | Control | ? | ? | + | + | ? | ? | ? | ? | ? | ? | ? | ? | ? | ? | ? | ? | ? | ? | ? |
| Sattler et al. (2019) | Multi-exercise group | + | ? | + | + | + | ? | ? | ? | + | + | + | + | + | + | + | N/A | ? | + | ? |
|  | Pedaling-Based Group | + | ? | + | + | + | + | ? | ? | + | + | + | + | + | + | + | N/A | ? | + | ? |
| Shabbir et al. (2017) | Functional Training | ? | ? | + | + | ? | ? | ? | ? | ? | ? | ? | ? | ? | ? | ? | ? | ? | ? | ? |
|  | Strengthening Training | ? | ? | + | + | ? | ? | ? | ? | ? | + | ? | ? | ? | ? | ? | ? | ? | ? | ? |
| Shanb et al. (2014) | Exercise + EMG | + | ? | + | + | ? | ? | + | + | + | ? | + | ? | + | + | + | N/A | ? | ? | ? |
|  | Exercise Only | ? | ? | + | + | ? | + | + | + | + | ? | + | ? | + | + | + | N/A | ? | ? | ? |
| Smith et al. (2019) | Fitness Tracker Group | + | ? | + | + | + | + | + | + | + | + | ? | ? | + | + | + | + | + | + | ? |
|  | Exercise Only | + | ? | + | + | + | + | + | + | + | + | ? | ? | + | + | + | + | + | + | ? |
| Stevens-Lapsley et al. (2012) | Standard + NMES | + | ? | + | + | ? | ? | + | + | + | + | + | + | + | + | + | N/A | ? | ? | + |
|  | Standard Care | + | ? | + | + | ? | ? | + | + | + | + | + | + | + | + | + | N/A | ? | ? | + |
| Tanaka et al. (2020) | Standard + Intensive Functional Rehab | + | ? | + | + | ? | ? | ? | ? | + | ? | ? | ? | + | ? | + | N/A | ? | ? | ? |
|  | Standard Care | ? | ? | ? | ? | ? | ? | ? | ? | ? | ? | ? | ? | ? | ? | + | N/A | ? | ? | ? |
| Tanaka et al. (2017) | Standard + Hybrid Assistive Limb | + | + | + | + | ? | ? | ? | ? | ? | ? | ? | ? | + | ? | + | N/A | ? | ? | + |
|  | Standard Care | ? | + | + | + | ? | ? | ? | ? | ? | ? | ? | ? | + | ? | + | N/A | ? | ? | + |
| Timmers et al. (2019) | Day-to-Day App Feedback | + | ? | + | + | ? | + | ? | ? | ? | + | + | ? | + | ? | + | N/A | ? | ? | + |
|  | 2x/week App Feedback | + | ? | + | + | ? | + | ? | ? | ? | ? | + | ? | + | ? | + | N/A | ? | ? | + |
| Tousignant et al. (2011) | Telehealth | + | ? | + | + | ? | ? | ? | ? | ? | ? | ? | ? | + | ? | ? | N/A | ? | ? | ? |
|  | Home Visit | ? | ? | + | + | ? | ? | ? | ? | ? | ? | ? | + | + | ? | ? | N/A | ? | ? | ? |
| Trudelle-Jackson et al. (2020) | Exercises + Step-Monitoring | + | ? | + | + | + | + | + | + | + | + | ? | ? | + | + | + | N/A | + | + | ? |
|  | Step-Monitoring | + | ? | + | + | ? | + | + | + | ? | + | ? | ? | + | + | + | N/A | + | ? | ? |
| Unver et al. (2016) | Weighted Exercises | + | ? | + | + | ? | ? | + | + | ? | + | ? | + | + | + | + | N/A | + | ? | ? |
|  | Non-weighted group | N/A | ? | + | + | ? | ? | + | + | ? | + | ? | + | + | + | + | N/A | + | ? | ? |
| Valdes et al. (2010) | Weighted Exercises | ? | ? | + | + | ? | ? | ? | ? | + | + | + | + | + | ? | + | N/A | ? | ? | ? |
|  | Standard care | ? | ? | + | + | ? | ? | ? | ? | + | + | ? | + | + | ? | + | N/A | ? | ? | ? |
| Valtonen et al. (2010) | Aquatic Resistance Group | + | ? | + | + | + | + | ? | + | ? | ? | ? | + | + | + | + | N/A | ? | + | ? |
|  | No Treatment | N/A | N/A | N/A | N/A | N/A | N/A | N/A | N/A | N/A | N/A | N/A | N/A | N/A | N/A | N/A | N/A | N/A | N/A | N/A |
| Vuorenmaa et al. (2014) | Monitored Home Exercise | + | ? | + | + | + | + | + | + | + | + | + | + | + | + | + | N/A | + | + | ? |
|  | Standard Care | ? | ? | + | + | + | ? | + | ? | ? | + | + | ? | + | ? | + | N/A | + | + | ? |
| Wozniak et al. (2017) | Standard Care + KTape | ? | ? | + | + | ? | ? | ? | ? | ? | ? | + | ? | + | ? | ? | ? | ? | ? | ? |
|  | Standard Care | ? | ? | + | + | ? | ? | ? | ? | ? | ? | ? | ? | + | ? | ? | ? | ? | ? | ? |
| Yousefian et al. (2017) | Experimental | + | ? | + | + | ? | ? | + | + | + | ? | ? | + | + | + | + | N/A | ? | ? | ? |
|  | Control | ? | ? | + | + | ? | ? | + | + | + | ? | ? | + | + | + | + | N/A | ? | ? | ? |
| Zietek et al. (2015) | Intensive Fast-Track | + | ? | + | + | ? | ? | ? | ? | + | ? | + | ? | + | ? | + | N/A | ? | ? | ? |
|  | Standard Track | ? | ? | + | + | ? | ? | ? | ? | ? | ? | + | ? | + | ? | + | N/A | ? | ? | ? |

**Key:** +: included; ?: not provided or not/insufficiently reported; N/A: Not applicable

| Intervention Group |  |
| --- | --- |
| Control Group |  |
| Studies with 3 Interventions |  |
