# Supplementary material for: Reporting of post-operative rehabilitation interventions for Total knee arthroplasty: a scoping review
Source: BMC Musculoskelet Disord. 2021 Jun 30;22:602. doi: 10.1186/s12891-021-04460-w (PMC8247251; doi:10.1186/s12891-021-04460-w)
Supplement: Supplementary file 6 — Additional file 6: Appendix F. Cochrane Risk of Bias Assessment. [file 12891_2021_4460_MOESM6_ESM.docx]

## Appendix F – Cochrane Risk of Bias Assessment

| **Author** | **Randomization** | **Allocation Concealment** | **Blinding** | **Incomplete Data** | **Selective Reporting** | **Other Sources of Bias** | **Evaluation of Risk of Bias** |
| --- | --- | --- | --- | --- | --- | --- | --- |
| Akbaba et al. (2016) | + | - | + | ? | - | + | High Risk of Bias |
| Alghadir et al. (2016) | + | - | + | + | ? | + | High Risk of Bias |
| Anneli et al. (2017) | + | - | + | ? | ? | + | High Risk of Bias |
| Aprile et al. (2011) | + | - | - | ? | ? | + | High Risk of Bias |
| Artz et al. (2017) | + | - | - | + | + | + | High Risk of Bias |
| Avramidis et al. (2011) | + | - | + | ? | ? | + | High Risk of Bias |
| Bade et al. (2017) | + | - | + | + | ? | + | High Risk of Bias |
| Bellelli et al. (2010) | ? | - | + | + | ? | - | High Risk of Bias |
| Bily et al. (2016) | + | - | - | + | ? | + | High Risk of Bias |
| Bini et al. (2017) | + | - | - | ? | ? | + | High Risk of Bias |
| Bohl et al. (2019) | + | - | - | + | + | + | High Risk of Bias |
| Brandes et al. (2018) | + | - | - | + | - | + | High Risk of Bias |
| Bruun-Olsen et al. (2013) | + | + | + | + | + | + | Low Risk of Bias |
| Bugbee et al. (2016) | ? |  |  | + | ? | + | High Risk of Bias |
| Buhaglar et al. (2017) | + | + | + | + | + | + | Low Risk of Bias |
| Bulthuis et al. (2007) | + | + | - | + | ? | + | High Risk of Bias |
| Cai et al. (2017) | + | - | - | + | ? | + | High Risk of Bias |
| Cai et al. (2018) | + | + | + | + | ? | + | Unclear Risk of Bias |
| Campbell et al. (2019) | + | - | + | + | + | + | High Risk of Bias |
| Chen et al. (2016) | + | + | + | + | ? | + | High Risk of Bias |
| Chen et al. (2017) | + | - | - | + | ? | + | High Risk of Bias |
| Christiansen et al. (2015) | + | + | + | ? | + | + | Unclear Risk of Bias |
| Christiansen et al. (2020) | + | + | + | ? | - | + | High Risk of Bias |
| De Fine et al. (2017) | ? | - | + | ? | ? | + | High Risk of Bias |
| Debbi et al. (2019) | + | + | + | + | ? | + | Unclear Risk of Bias |
| Demircioglu et al. (2015) | + | - | - | + | ? | + | High Risk of Bias |
| den Hertog et al. (2012) | + | + | - | + | ? | + | High Risk of Bias |
| Doerfler et al. (2016) | ? | + |  | + | ? | + | Unclear Risk of Bias |
| Donec & Krisciunas (2014) | + | + | - | + | ? | + | High Risk of Bias |
| Ebert et al. (2013) | + | + |  | + | ? | + | Unclear Risk of Bias |
| Eichler et al. (2019) | + | - | - | + | - | + | High Risk of Bias |
| Eisermann et al. (2004) | ? | - | - | + | ? | - | High Risk of Bias |
| Evgeniadis et al. (2008) | + | + | + | + | ? | + | Unclear Risk of Bias |
| Ficklscherer et al. (2016) | ? |  |  | ? | ? | + | High Risk of Bias |
| Fleischman et al. (2019) | + | + | + | + | + | + | Low Risk of Bias |
| Fortuno Godes et al. (2010) | + | - | - | ? | ? | + | High Risk of Bias |
| Fransen et al. (2017) | + | + | + | + | - | + | High Risk of Bias |
| Frost et al. (2002) | + | - | + | + | ? | + | High Risk of Bias |
| Fung et al. (2012) | + | - | + | + | + | + | High Risk of Bias |
| Gianola et al. (2020) | + | + | + | + | + | + | Low Risk of Bias |
| Giaquinto et al. (2010) | + | + | + | ? | ? | + | Unclear Risk of Bias |
| Han et al. (2015) | + | + | + | + | - | + | High Risk of Bias |
| Hardt et al. (2018) | + | + | + | + | ? | + | Unclear Risk of Bias |
| Harikesavan et al. (2017) | + | - | + | ? | ? | + | High Risk of Bias |
| Harmer et al. (2009) | + | + | + | + | ? | + | Unclear Risk of Bias |
| Hepperger et al. (2017) | + | - | - | ? | ? | + | High Risk of Bias |
| Hoorntje et al. (2020) | ? | - | - | ? | - | + | High Risk of Bias |
| Husby et al. (2018) | + | - | - | + | - | + | High Risk of Bias |
| Iwakiri et al. (2020) | + | + | + | + | - | + | High Risk of Bias |
| Jakobsen et al. (2014) | + | + | + | + | + | + | Low Risk of Bias |
| Jin et al. (2018) | ? | - | - | ? | ? | - | High Risk of Bias |
| Jogi et al. (2015) | + | + | + | ? | - | + | High Risk of Bias |
| Johnson et al. (2010) | + | - | - | ? | ? | + | High Risk of Bias |
| Ju et al. (2019) | + | - | - | ? | ? | - | High Risk of Bias |
| Karaman et al. (2017) | ? | - | - | + | ? | + | High Risk of Bias |
| Kauppila et al. (2010) | + | + | - | + | ? | + | High Risk of Bias |
| Kelly et al. (2016) | + | + | + | + | - | + | High Risk of Bias |
| Kramer et al. (2003) | - | - | + | + | - | - | High Risk of Bias |
| Lee et al. (2020) | + |  | - | + | ? | + | High Risk of Bias |
| Lenguerrand et al. (2019) | + | + | - | + | + | + | High Risk of Bias |
| Lenssen et al. (2006) | + | ? |  | + | ? | + | Unclear Risk of Bias |
| Levine et al. (2013) | + | - | - | + | ? | + | High Risk of Bias |
| Li et al. (2019) | + | + | ? | + | ? | + | Unclear Risk of Bias |
| Liao et al. (2013) | + | + | + | + | ? | + | Unclear Risk of Bias |
| Liao et al. (2020) | + | + | + | + | + | + | Low Risk of Bias |
| Liebs et al. (2010) | + | + | ? | + | + | + | Unclear Risk of Bias |
| Liebs et al. (2012) | + | + | ? | + | ? | + | Unclear Risk of Bias |
| Lin et al. (2018) | + | + | - | + | ? | + | High Risk of Bias |
| Lowe et al. (2012) | + | + | + | + | + | + | Low Risk of Bias |
| Lysack et al. (2005) | ? | - | - | + | ? | + | High Risk of Bias |
| Madsen et al. (2013) | ? | + | + | ? | + | + | Unclear Risk of Bias |
| Mahomed et al. (2008) | + | - | - | + | ? | + | High Risk of Bias |
| McAvoy (2009) | + | + | + | + | ? | + | Unclear Risk of Bias |
| Mitchell et al. (2005) | + | + | + | + | ? | + | Unclear Risk of Bias |
| Mockford et al. (2008) | + | - | + | + | ? | - | High Risk of Bias |
| Moffet et al. (2004) | + | + | + | + | ? | - | High Risk of Bias |
| Moffet et al. (2015) | + | + | + | + | ? | - | High Risk of Bias |
| Monticone et al. (2013) | + | + | + | + | ? | + | Unclear Risk of Bias |
| Moutzouri et al. (2018) | + | + | + | + | + | + | Low Risk of Bias |
| Munin et al. (1998) | + | + | + | + | ? | - | High Risk of Bias |
| Ko et al. (2013) | + | + |  | + | + | + | Low Risk of Bias |
| Paravlic et al. (2019) | + | + | - | + | + | + | High Risk of Bias |
| Paxton et al. (2018) | ? | - | - | + | + | + | High Risk of Bias |
| Peiris et al. (2012) | + | + | + | + | + | + | Low Risk of Bias |
| Petterson et al. (2009) | ? | - | + | + | ? | + | High Risk of Bias |
| Piqueras et al. (2013) | + | - | + | + | ? | + | High Risk of Bias |
| Piva et al. (2010) | + | ? | ? | + | + | + | Unclear Risk of Bias |
| Piva et al. (2017) | + | + | + | + | + | + | Low Risk of Bias |
| Piva et al. (2019) | + | + | + | + | + | + | Low Risk of Bias |
| Prvu Bettger et al. (2020) | + | + | - | + | + | + | High Risk of Bias |
| Rahmann et al. (2009) | + | + | + | + | + | + | Low Risk of Bias |
| Rajan et al. (2004) | + | - | + | - | ? | - | High Risk of Bias |
| Roig-Casas√∫s et al. (2018) | + | ? | + | + | ? | + | Unclear Risk of Bias |
| Russell et al. (2011) | + | + | + | + | + | + | Low Risk of Bias |
| Russo et al. (2017) | + | ? | + | - | ? | + | High Risk of Bias |
| Sattler et al. (2019) | + | + | + | + | + | + | Low Risk of Bias |
| Shabbir et al. (2017) | + | + | + | - | ? | - | High Risk of Bias |
| Shanb et al. (2014) | + | + | - | + | ? | + | High Risk of Bias |
| Smith et al. (2019) | + | - | - | + | ? | + | High Risk of Bias |
| Stevens-Lapsley et al. (2012) | + | - | - | + | ? | + | High Risk of Bias |
| Tanaka et al. (2017) | + | - | + | + | ? | + | High Risk of Bias |
| Tanaka et al. (2020) | + | + | + | + | + | + | Low Risk of Bias |
| Timmers et al. (2019) | + | + | - | + | + | + | High Risk of Bias |
| Tousignant et al. (2011) | + | + | - | + | - | + | High Risk of Bias |
| Trudelle-Jackson et al. (2020) | + | + | - | + | ? | + | High Risk of Bias |
| Unver et al. (2016) | + | - | - | + | ? | + | High Risk of Bias |
| Valdes et al. (2010) | - | - | - | - | ? | + | High Risk of Bias |
| Valtonen et al. (2010) | ? | + | + | + | + | + | Unclear Risk of Bias |
| Vuorenmaa et al. (2014) | + | ? | + | + | + | + | Unclear Risk of Bias |
| Woźniak-Czekierda et al. (2017) | ? | - | - | + | ? | - | High Risk of Bias |
| Yousefian et al. (2017) | + | ? | + | + | ? | + | Unclear Risk of Bias |
| Zietek et al. (2015) | + | + | + | + | ? | + | Unclear Risk of Bias |

**Key:**

| + = low risk of bias | - = high risk of bias | ? = unclear risk of bias |
| --- | --- | --- |
